# PLIN5 phosphorylation orchestrates mitochondria lipid-droplet coupling to control hepatic lipid flux and steatosis

**DOI:** 10.1038/s42255-026-01476-1

**Published:** 2026-03-23

**Authors:** Sun Woo Sophie Kang, Lauryn A. Brown, Colin B. Miller, Katherine M. Barrows, Jihye L. Golino, Hanyang Liu, Constance M. Cultraro, Daniel Feliciano, Mercedes B. Cornelius-Muwanuzi, Kirsten Remmert, Jonathan M. Hernandez, Andy D. Tran, Michael Kruhlak, Alexei Lobanov, Maggie Cam, Natalie Porat-Shliom

**Affiliations:** 1 Cell Biology and Imaging Sections, Thoracic and GI Malignancies Branch, Center for Cancer Research, National Cancer Institute, National Institutes of Health, Bethesda, MD USA; 2 Surgical Oncology Program, Center for Cancer Research, National Cancer Institute, National Institutes of Health, Bethesda, MD USA; 3 Cavendish Laboratory, Department of Physics, University of Cambridge, J. J. Thomson Avenue, Cambridge, UK; 4 Laboratory of Cancer Biology and Genetics, Center for Cancer Research, National Cancer Institute, National Institutes of Health, Bethesda, MD USA; 5 CCR Collaborative Bioinformatics Resource, National Cancer Institute, National Institutes of Health, Bethesda, Maryland USA

**Keywords:** Mitochondria, Fat metabolism, Metabolism

## Abstract

Steatotic liver disease is common, yet the mechanisms by which hepatocytes cope with surges in dietary fatty acids remain unclear. Here we use single-cell tissue imaging (scPhenomics) and spatial proteomics to map lipid handling across dietary states. Fasting remodeled mitochondria and lipid droplets (LDs), increasing mitochondria–LD contacts, whereas contacts were infrequent in Western diet (WD)-fed male mice. Fasting also elevated perilipin-5 (PLIN5), a mediator of mitochondria-LD tethering. PLIN5 overexpression modulated contact formation in a phosphorylation-dependent manner: the S155A variant enhanced organelle contacts and LD expansion, whereas the S155E variant reduced contacts and yielded fewer, smaller LDs. Overexpression of the S155A variant in WD reduced lipotoxicity. These data reveal an adaptive organelle-interaction program that channels lipids during nutrient stress and is attenuated by an obesogenic diet. Our work establishes scPhenomics for spatially resolved cell-state analysis and identifies PLIN5 phosphorylation as a lever to tune hepatocyte lipid flux, suggesting therapeutic potential for targeting mitochondria–LD coupling.

## Abstract (web summary)

Using single-cell tissue imaging and spatial proteomics to study mitochondria–lipid droplet coupling in hepatocytes, PLIN5 phosphorylation is identified as a mediator of lipid flux and nutrient stress responses.

## Main

Steatotic liver disease, caused by excessive hepatic lipid buildup, is a common condition affecting about one-third of adults worldwide^1^. Interestingly, fasting also induces lipid buildup in the liver owing to increased fatty acid (FA) transport from adipose tissue and a concurrent reduction in very-low-density lipoprotein (VLDL) secretion^2,3^. However, it remains unclear how hepatocytes manage this lipid influx during nutrient deprivation, and whether these mechanisms can be harnessed to restore function in steatosis.

The liver plays a central role in regulating lipid metabolism, alternately promoting lipid oxidation and synthesis depending on nutrient availability. Following a meal, lipids produced by the liver are sent to adipose tissue for storage. During fasting, FAs stored in adipose tissue are mobilized to the liver, where they are used to synthesize alternative fuel sources^4^. Notably, in homeostasis, lipid metabolism is spatially segregated in the liver; periportal hepatocytes are primarily involved in lipid oxidation, and pericentral hepatocytes are associated with lipid synthesis^5,6^. How these populations of hepatocytes adapt to the influx of lipids observed during fasting and disease remains poorly understood.

Metabolic flexibility, defined as the ability to sense and respond to changes in nutrient availability, is crucial for regulating adaptive organ responses^2,7^. In hepatocytes, this flexibility depends on the rapid structural reorganization of organelles, which form interconnected networks known as metabolic sub-compartments. For example, the remodeling of mitochondrial–LD contacts facilitates lipid transfer either for oxidation in the mitochondria or storage in LD, depending on the cell and tissue type^8,9^. Perilipin 5 (PLIN5) is one of several proteins that facilitates mitochondria–LD interactions^10,11^. It localizes to LDs and recruits mitochondria through a unique carboxy-terminal region^12–14^. Although mitochondrial and LD organization is remodelled in liver physiology and disease, its functional outcomes remain unclear, partly because studying these processes in tissues is challenging.

In this study, we examined the effects of increased hepatic lipid flux on organelle organization, focusing on mitochondria and LDs—key organelles that form contact sites to regulate lipid metabolism^10,15^. Using a novel single-cell phenotypic profiling (scPhenomics) approach across different nutritional states, we discovered that mitochondria–LD contact sites are upregulated throughout the lobules of fasted mice, but not in mice fed a short-term WD to mimic steatotic liver disease. Combining this technique with spatial proteomics, we identified the upregulation of PLIN5. In vivo hepatocyte-specific overexpression of PLIN5 promoted the formation of mitochondria–LD contact sites in a phosphorylation-dependent manner and increased triglyceride (TG) storage in LDs. During short-term WD feeding, the formation of mitochondria–LD contacts protected hepatocytes from lipotoxicity by channeling potentially harmful free fatty acids (FFAs) into LDs. Consistently, prolonged exposure to a WD, in both mouse and human samples, was associated with increased abundance of mitochondria–LD interactions. These findings suggest that the dynamic assembly and disassembly of mitochondria–LD contact sites play a pivotal role in hepatic lipid management and could be a therapeutic target.

## Results

### Mitochondria and LD topologies mirror the spatial zonation of hepatic lipid utilization and synthesis

**Fig. 1:**
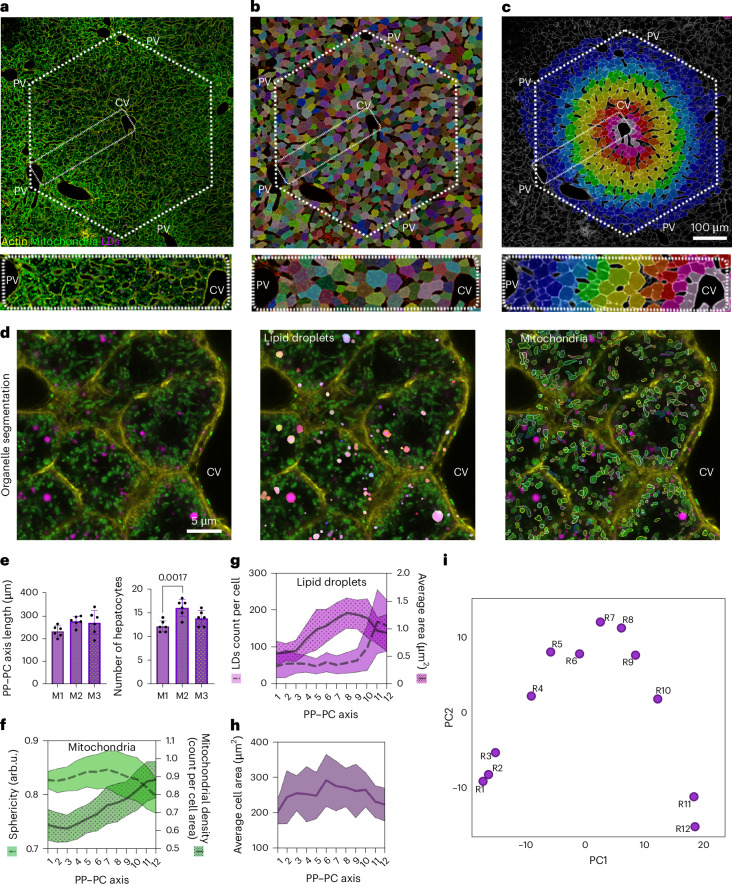
LD and mitochondrial topology define zones in the lobule. **a**, Confocal image of a liver lobule from mtDendra2 (green) transgenic mouse. Actin was labelled with phalloidin (yellow), and lipid droplets with LipidTox (magenta). A representative PP–PC axis is marked with a dotted rectangle. **b**, Segmentation of hepatocytes, guided by actin and mitochondria labelling. PV, portal vein; CV, central vein. **c**, Colour map representing the spatial positioning of hepatocytes on the basis of their distance from the central vein. **d**, Segmentation of LDs and mitochondria in cells. **e**, Quantification of PP–PC axis length and the number of hepatocytes per axis. Data are presented as mean ± s.d. from six PP–PC axes analysed from three mice (Supplementary Data 1). Statistical significance was calculated using a two-tailed unpaired Student’s *t*-test. *P* = 0.0017. **f**,**g**, Quantification of morphological features, including mitochondrial sphericity and mitochondrial density (**f**), and LD count and LD area (**g**). **h**, Quantification of average hepatocyte cell area across the PP–PC axis. **i**, Principal component analysis plot of hepatocyte (R1–R12) clustering. Data are presented as mean ± s.d. Source data

**Extended Data Fig. 1:**
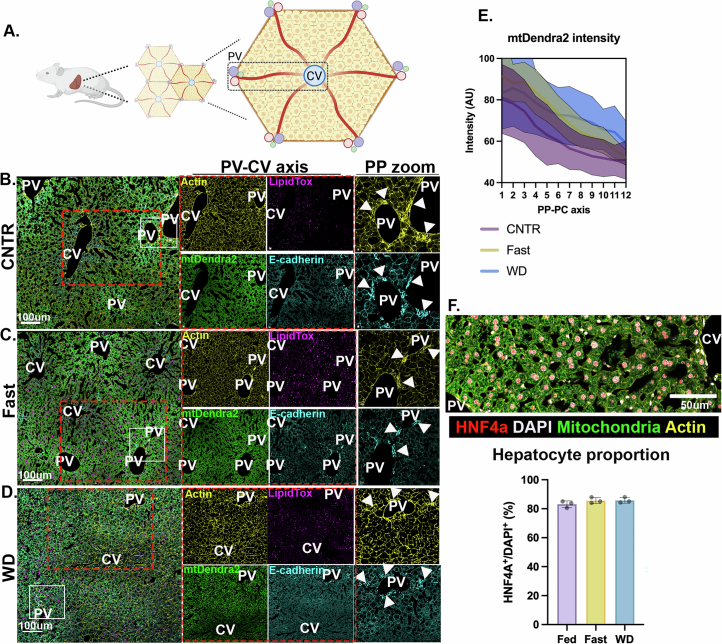
Anatomical landmarks guide tissue orientation in scPhenomics. **(A)** Anatomical organization of the mouse liver lobule. The model was created with Biorender.com. Representative confocal images of whole lobule (left), PP-PC axes (middle), and close-up on PP hepatocytes (right), from mtDendra2 transgenic mice fed a control (CNTR) diet **(B)**, overnight fasted **(C)**, or Western Diet (WD) **(D)**. Mitochondria are shown in green, actin was labeled with phalloidin (yellow), LDs were labeled with LipidTox (magenta), and the PP marker E-cadherin is shown in cyan. Arrowheads point at actin-rich structures in the portal regions that also label with E-cadherin. Scale bar 100 μm. **(E)** mtDendra2 fluorescence intensity is shown across space and dietary conditions. Data is presented as mean ± SD from 18 PP-PC axes analyzed from three mice (Supplementary data 1). **(F)** Liver sections from Dendra2 mice fed, fasted, or WD-fed were labeled with DAPI (white), HNT4a (red), and actin (yellow). Scale bar 50 μm. The proportion of hepatocytes (HNF4a^+^/DAPI^+^ cells) across three PP-PC axes from two independent experiments is presented as mean ± SD. PV: portal vein; CV: central vein; PP: periportal; PC: pericentral; LD: Lipid droplets.

Hepatocytes are organized along sinusoids in the lobule, the functional unit of the liver (Extended Data Fig. 1a). Recent studies, including our own, have demonstrated that mitochondrial morphology, LD content and lipid metabolism vary across the periportal–pericentral axis (PP–PC)^16–18^. We mapped organelle topology at single-cell resolution to further investigate the relationship between organelle structure and lipid metabolism. To this end, confocal images of liver sections from transgenic mice^19^ expressing mitochondrial-targeted Dendra2 (mtDendra2)^19^ stained with BODIPY and phalloidin to mark LDs and cell structure, respectively, were acquired (Fig. 1a). The orientation of the PP–PC axis was determined using actin labelling of the bile ducts in the portal triad (Extended Data Fig. 1b–d). In addition, Dendra2 fluorescent intensity was consistently higher in PP regions, providing an indirect landmark for tissue orientation (Extended Data Fig. 1e).

We extracted cellular and organelle features using a custom Python-based workflow (Python v3.10) that uses deep-learning segmentation^20^. The segmented hepatocytes (Fig. 1b) were assigned a spatial coordinate (a value from 0 to 1) by calculating their distance from the central vein and dividing it by the total PP–PC length (Fig. 1c). The PP–PC axes were manually cropped (dashed rectangle). Similarly, we segmented organelles and measured geometrical parameters (such as area, density, sphericity; see Methods) using the region props function in the scikit-image^21^ (Fig. 1d). Analysis of 18 PP–PC axes from three mice showed an average axis length of 250 µm, comprising 12–16 cells per axis (Fig. 1e). To ensure that there was at least one hepatocyte per bin, we divided the PP–PC axes into 12 bins; bin 1 was the closest to the portal vessels, and bin 12 was closest to the central vein. More than 80% of the cells analysed in the segmented regions were HNF4α hepatocytes (Extended Data Fig. 1f).

We plotted representative mitochondrial and LD features across the PP–PC axis (Supplementary Data 1). Mitochondria in portal and mid-lobular hepatocytes had higher sphericity (a value of 1 indicates a perfect sphere) and lower density. By contrast, pericentral hepatocytes (bins 9–12) exhibited lower sphericity, indicating a more tubular morphology, along with increased mitochondrial density (Fig. 1f). LDs were sparse and small in the portal regions (bins 1–3). A lower LD abundance persisted through bin 9, followed by a sharp increase in bins 10–12, reflecting the higher LD content in pericentral hepatocytes (Fig. 1g). The average LD area gradually increased from bins 3–9 before slightly decreasing in pericentral regions, where LD numbers were the highest (bins 10–12; Fig. 1g).

**Extended Data Fig. 2:**
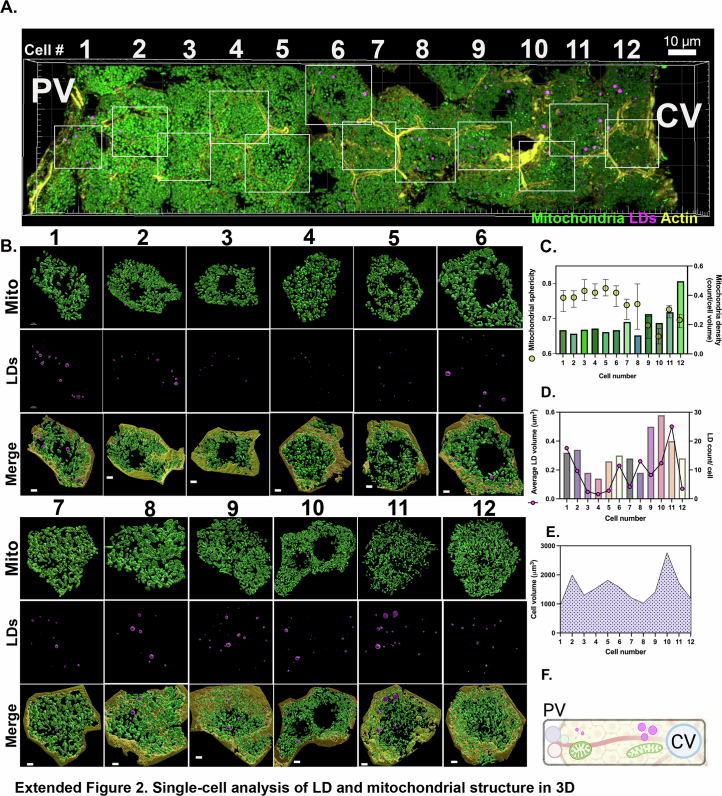
Single-cell analysis of LD and mitochondrial structure in 3D. **(A)** Representative projection of a z-stack from mtDendra2 mice showing mitochondria (green), LDs (magenta), and actin (yellow). Scale bar 10 μm. **(B)** 3D surfaces depicting mitochondria (green), LDs (magenta), and actin (yellow) at single-cell resolution. Cells are numbered 1 to 12 along the PP-PC axis. Scale bar 2 μm **(C)** Analysis of mitochondrial sphericity and density. Data presented as mean ± SD from one representative cell. **(D)** Analysis of LD volume and count. **(E)** Analysis of cell volume. **(F)** Schematic representation of mitochondria and LDs phenotypes along the PP-PC axis. The schematic was created with Biorender.com. A representative lobule is shown; similar trends were observed in 3 independent experiments. PV: portal vein; CV: central vein; LD: lipid droplet. Source data

To validate that measurements from two-dimensional (2D) sections accurately represent the three-dimensional (3D) morphology of organelles, we also assessed mitochondrial and LD features in confocal *Z*-stacks (Extended Data Fig. 2). We manually segmented individual hepatocytes and rendered cell and organelle surfaces using Imaris software (Extended Data Fig. 2a,b). We observed an inverse relationship between mitochondrial sphericity and density in 2D, which was confirmed in 3D (Extended Data Fig. 2c). Similarly, LD features were zonated along the PP–PC axis in both the 2D and 3D datasets (Extended Data Fig. 2d). Dimensionality reduction had no impact on organelle features, showing similar trends in both 2D and 3D. Cell area in the 2D and 3D datasets is shown in Fig. 1h and Extended Data Fig. 2e. An illustration summarizing the organelle features observed in three independent experiments is shown in Extended Data Fig. 2f.

Next, we examined whether the combination of topological features encoded spatial signatures. Principal component analysis (PCA) was performed by integrating the mitochondrial and LD features in Supplementary Data 1. The analysis showed that neighbouring hepatocytes cluster together (Fig. 1i). Each PCA dot represents the averaged mitochondrial and LD features for each bin or cell along the PP–PC axis (R1–R12). Three clusters emerged: R1–R3, R4–R10 and R11–R12. The inclusion of neighbouring cells in clusters could reflect overlapping functions (Fig. 1i).

Overall, the single-cell phenotypic profiling (scPhenomics) of mitochondria and LDs efficiently used 2D datasets, allowing faster data acquisition, larger sample sizes and automated analysis. This novel scPhenomics approach recapitulates spatial signatures associated with the functional dichotomy of lipid oxidation versus synthesis and storage, potentially defining the hepatic zones responsible for lipid handling.

### Increased lipid flux rewires organelle circuits in the zonated liver

**Fig. 2:**
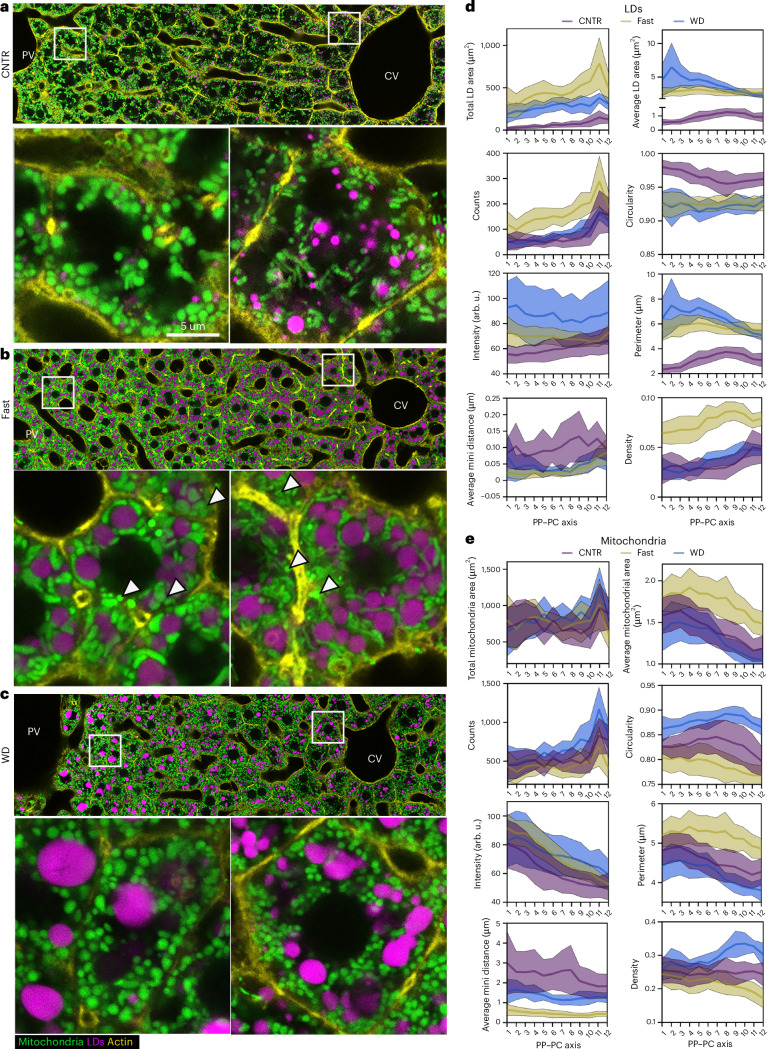
Increased lipid flux induces rewiring of LD and mitochondria circuits. **a**–**c**, Representative confocal images of PP–PC axes from mtDendra2 transgenic mice fed control (CNTR) diet (**a**), overnight fasted (**b**) or WD (**c**). Mitochondria are shown in green, actin was labelled with phalloidin (yellow) and lipid droplets were labelled with LipidTox (magenta). For each condition, representative PP and PC cells are shown in enlarged insets. **d**,**e**, LD (**d**) and mitochondria (**e**) features, including area (total and average per cell), count, circularity, intensity, perimeter, minimal distance and density, are shown across space and dietary conditions. scPhenomics data are presented as mean ± s.d. from 18 PP–PC axes analysed from three mice (Supplementary Data 1). PP, periportal; PC, pericentral.

To examine how increased hepatic lipid influx impacts organelle features, we subjected mtDendra2 mice to different dietary conditions: control diet (CNTR), fasting overnight (16 h) or fed a WD for 4 weeks. Liver sections were stained with BODIPY and phalloidin to label LDs and cell structure, respectively, followed by scPhenomics workflow analysis (Fig. 2a–c and Supplementary Data 1).

Fasting leads to hepatic lipid accumulation due to increased lipid influx from adipose tissue. As expected, LD content increased across the entire lobule (Fig. 2b). Although LD size remained relatively uniform, the number of LDs was higher in pericentral (PC) hepatocytes. Mitochondria in periportal (PP) and mid-lobular regions appeared longer and thicker (Fig. 2b, insets). Notably, long and thick mitochondria frequently wrapped around LDs, forming extensive contact sites between the two organelles (Fig. 2b, insets). However, a subset of mitochondria in each hepatocyte retained spherical morphology and did not interact with LDs (Fig. 2b, arrowheads).

**Extended Data Fig. 3:**
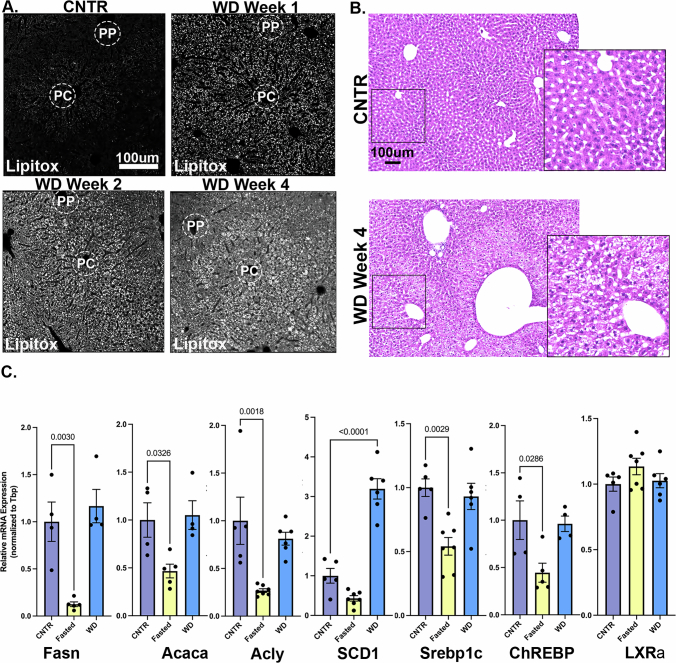
Hepatic steatosis and DNL in short-term WD feeding. **(A)** Confocal images of liver sections from C57BL/6 J mice fed either a CNTR or WD, as indicated. LDs were labeled with LipidTox (white). Scale bar 100 μm. **(B)** Liver sections from mice fed either CNTR or WD for 4 weeks stained with H&E. Scale bar 100 μm. **(C)** Relative expression levels of lipogenesis enzymes (*Fasn*, *Acaca*, *Acly*, *Scd1*) and transcription factors (*Srebp1c*, *ChREBP*, and *LXRa*) in control diet (CNTR), fasted, or 4-week WD-fed mice. Data are presented as mean ± SD from n = 5 independent experiments. Statistical significance was determined using a one-way ANOVA, with a Bonferroni post hoc test was employed to correct for multiple comparisons. LD: lipid droplets; PP: periportal; PC: pericentral; CNTR: control; WD: Western diet; DNL: de novo lipid synthesis. Source data

Dietary lipid influx also leads to hepatic lipid accumulation in WD-fed mice. Four weeks of WD consumption was sufficient to induce simple steatosis (Extended Data Fig. 3a) without causing lobular inflammation or hepatocellular ballooning (Extended Data Fig. 3a). To rule out the possibility that de novo lipogenesis (DNL) had been activated^22,23^, we examined the expression of DNL enzymes and transcription factors (Extended Data Fig. 3c). Increased lipid influx due to fasting led to transcriptional inhibition, as previously reported^23^. However, 4 weeks of WD feeding had no effect on expression levels, suggesting that steatosis in WD-fed mice was due to esterification of dietary lipids to form LDs. One exception was the expression levels of stearoyl-CoA desaturase 1 (SCD1) (Extended Data Fig. 3c). SCD1 activity is crucial for TG packaging and LD expansion^24,25^, so the upregulation of *Scd1* in WD-fed mice could be an adaptive response to lipid excess.

Although WD-fed mice accumulated LDs throughout the lobule, the size, number and distribution of these LDs differed markedly from those in fasted mice (compare Fig. 2b,c). Specifically, LDs in PP and mid-lobular hepatocytes were larger than those in PC regions. The WD-fed mice had more spherical mitochondria with reduced LD interactions (Fig. 2c). Overall, increased exogenous lipid flux, whether through fasting or WD, triggered distinct remodeling of both LDs and mitochondria. Employing scPhenomics allowed for precise quantification of organelle features across different zones and dietary conditions (Fig. 2d,e). Notably, most features were influenced by both the spatial positioning of the cells and the dietary intervention. This supports the notion that LD and mitochondrial features encode spatial morphological signatures of zonation, providing insights into the cellular metabolic state.

**Fig. 3:**
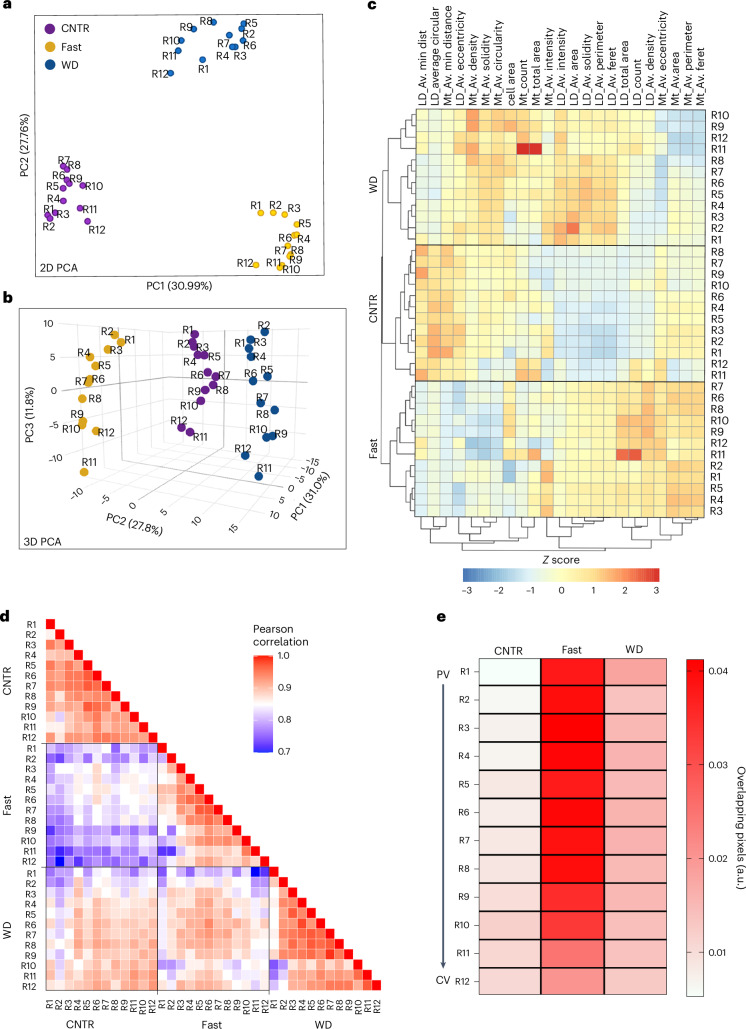
scPhenomics informs of cellular states and zonation of lipid metabolism. **a**,**b**, PCA plots of single-cell organelle features (R1–R12) under different dietary conditions in 2D (**a**) and 3D (**b**). **c**, Heatmap showing single-cell (R1–R12) organelle features under different dietary conditions. **d**, Correlation matrix heatmap of organelle features under different dietary conditions. Mt, mitochondria; LD, lipid droplet; Av, average; feret, longest distance between any two boundary points. **e**, Heat map of mitochondria and LDs overlapping pixels across the PP–PC axis (R1–R12) and diets. All plots were created with data in Supplementary Data 1.

Next, we integrated topological features from mitochondria and LDs to assess how spatial signatures changed in response to dietary perturbation. When plotted in a 2D PCA space, hepatocytes from CNTR, fasted and WD-fed mice formed tightly packed clusters, highlighting the impact of dietary state on organelle topology (Fig. 3a). Incorporating a third component into the analysis further increased the spatial diversity in each group. This demonstrates the distinct remodelling of organelles that depends on both the spatial positioning of the cell and the metabolic state (Fig. 3b). We used a dendrogram combined with a heatmap to further examine the information encoded by organelle features. This approach involves unsupervised clustering to group mitochondria and LD average features on the basis of similarity. The resulting heatmaps reveal distinct spatial patterns of organelle features under different nutritional conditions. CNTR-fed hepatocytes clustered into roughly three groups, consistent with the PCA data and potentially representing three zones. By contrast, fasted and WD-fed hepatocytes clustered into at least two groups of neighbouring cells (Fig. 3c).

To examine how individual cells along the PP–PC axis (R1–R12) respond to either fasting or a WD, Pearson’s correlation coefficient was calculated, and the pairwise correlations are shown in a heatmap (Fig. 3d). Compared with organelles from CNTR-fed cells, fasted organelles showed low correlation coefficient values (light–dark purple), indicating that fasting induces extensive remodelling of LD and mitochondria architecture. Conversely, organelle features from WD-fed livers exhibited a high correlation with the corresponding features from the CNTR diet, indicating low organelle remodelling in livers of WD-fed mice. However, a subset of PP hepatocytes (R1–R3) was affected by the WD, as demonstrated by the low coefficient values when compared with CNTR.

Of the features we measured, mitochondria–LD contacts were impacted by fasting and WD (Fig. 2). The distribution of mitochondria–LD contacts across the lobule and diet is shown in Fig. 3e. In the CNTR diet, LD content was low and localized to PC hepatocytes (Fig. 1g), consistent with the absence of mitochondria–LD overlapping pixels in PP and mid-lobular regions (R1–R8), and relatively low pixel overlap in PC regions. Conversely, fasting dramatically increased the overlapping pixels throughout the lobule, except for R11 and R12 in PC regions. By contrast, WD-fed mice exhibited only a modest increase in overlapping pixels, with values similar to those measured in PC regions of CNTR-fed mice (Fig. 3e). The heightened LD–mitochondria interactions in response to fasting and the reduction of these interactions in WD-fed mice were relatively uniform across the lobule.

### Comparative proteomics of sorted hepatocytes from fed and fasted mice

**Extended Data Fig. 4:**
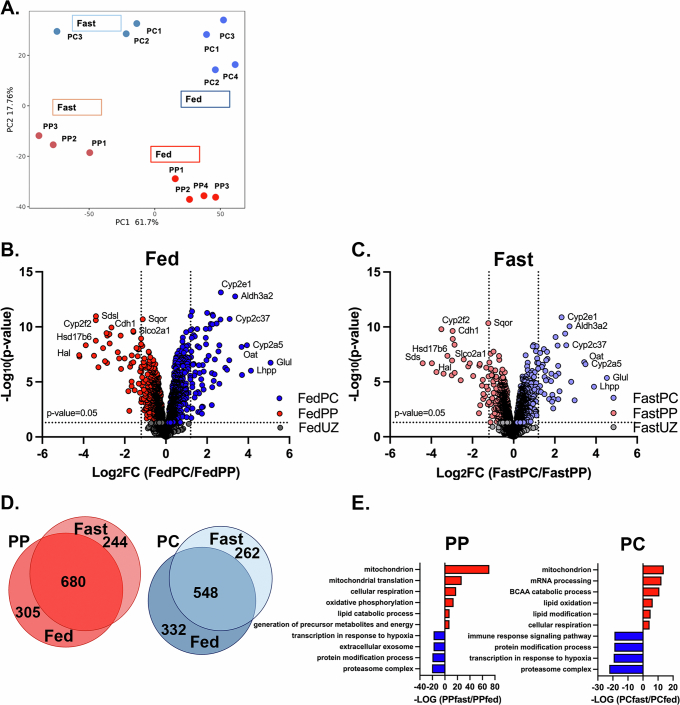
Comparative proteomics of spatially sorted hepatocytes. **(A)** Principal component analysis (PCA) of spatially sorted hepatocytes from fed (n = 4 mice) and fasted mice (n = 3 mice). Volcano plot showing the PC to PP log2 fold-change (x-axis) and the -log10 p-value (y-axis) for identified proteins in fed **(B)** and fasted **(C)**. Statistical significance was calculated using a two-tailed unpaired Student’s *t*-test. PP and PC proteins are shown in red and blue in fed, or light red and light blue in the fasted mice, respectively. **(D)** Venn diagram illustrating PP or PC signature proteins in fed and fasted mice. **(E)** GO enrichment analysis for the proteomics data in the spatially sorted cells.

To identify candidates that mediate mitochondria–LD interactions, we performed spatial proteomics. PP and PC hepatocytes were enriched from four ad-lib-fed CNTR and overnight-fasted mice using surface markers and fluorescence-activated cell sorting (FACS, as previously described^17^). The sorted hepatocytes and unsorted total control were subjected to tandem mass tag (TMT)-based quantitative mass spectrometry for total proteome analysis. Out of 4,999 identified proteins, 1,865 (37%) in CNTR-fed mice and 1,734 (35%) in fasted mice exhibited zonation (Supplementary Data 2). PCA analysis showed clustering of the spatially sorted hepatocytes is influenced by their lobular position (PP or PC) and the diet (fed or fasted) (Extended Data Fig. 4a). Plotting of the PC to PP ratios in CNTR-fed or fasted hepatocytes shows that fasting did not influence hepatocyte sorting (Extended Data Fig. 4b,c).

**Fig. 4:**
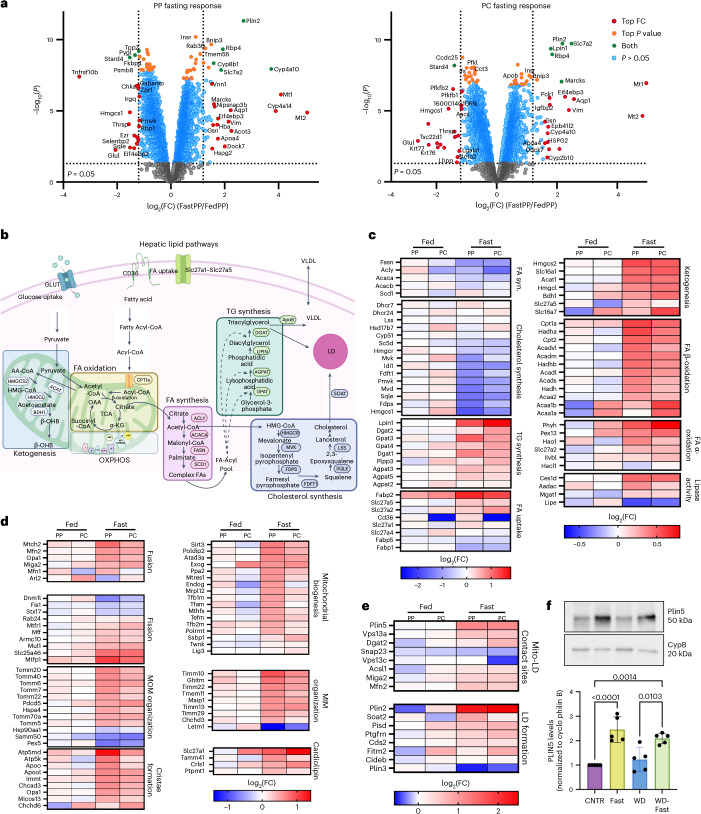
Fasting-induced remodelling of the hepatic proteome in spatially sorted hepatocytes. **a**, Volcano plot showing the PP or PC, fast-to-fed log_2_(fold change (FC)) and the –log_10_(*P*) for identified proteins. Colour coding highlighting significance on the basis of *P* value and fold change. Proteomics data were analysed using the Limma R package (version 3.40.6). **b**, Lipid pathways in hepatocytes. OXPHOS, oxidative phosphorylation. **c**–**e**, Heatmaps of proteins involved in lipid metabolic pathways (**c**), mitochondrial dynamics and structure (**d**), mitochondria–LD interactions and LD formation (**e**) across PP and PC sorted hepatocytes from fed and fasted mice. Heat map values were derived from the means of three independent experiments and normalized to the total protein expression in livers from fed mice. **f**, Western blot analysis of PLIN5 levels under different dietary conditions. Data are presented as mean ± s.d. from *n* = 5 independent experiments. Statistical significance was calculated using a two-tailed unpaired Student’s *t*-test. Schematic in **b** created in BioRender; Porat-Shliom, N. https://BioRender.com/3ps9u0r (2026). Source data

The fasting response in the two hepatocyte populations was examined using volcano plots (Fig. 4a). We observed considerable overlap between PP and PC hepatocytes in the sets of proteins that were upregulated as well as those that were downregulated. For example, the mitophagy receptor BNIP3, which is enriched in PC regions^17,26,27^, was upregulated in both PP and PC hepatocytes during fasting, consistent with increased mitophagy under these conditions. Notably, more than half of the PP and PC proteomes remained unchanged in response to fasting, suggesting that zonation is largely preserved during nutritional stress, with only a subset of the proteome undergoing remodelling (Extended Data Fig. 4d). Pathway analysis revealed that many regulated pathways are common to PP and PC hepatocytes and were linked to mitochondrial function and lipid metabolism (Extended Data Fig. 4e).

In mice fed an ad libitum CNTR diet, many hepatic lipid pathways (summarized in Fig. 4b) are zonated, as shown by the non-uniform expression of key enzymes (Fig. 4c). In response to fasting, lipid pathway proteins were either upregulated or downregulated throughout the lobule (Fig. 4c compares PP and PC in the fasted state). As expected, the expression of proteins involved in FA and cholesterol synthesis was largely reduced during nutrient deprivation. However, proteins involved in TG synthesis, an anabolic process that paradoxically increases during fasting^28^, were an exception, likely to facilitate the temporary storage of the excess free FA in LDs to prevent lipotoxicity. Additionally, proteins involved in catabolic processes, such as FA lipolysis, oxidation and ketogenesis, were also upregulated (Fig. 4c).

**Extended Data Fig. 5:**
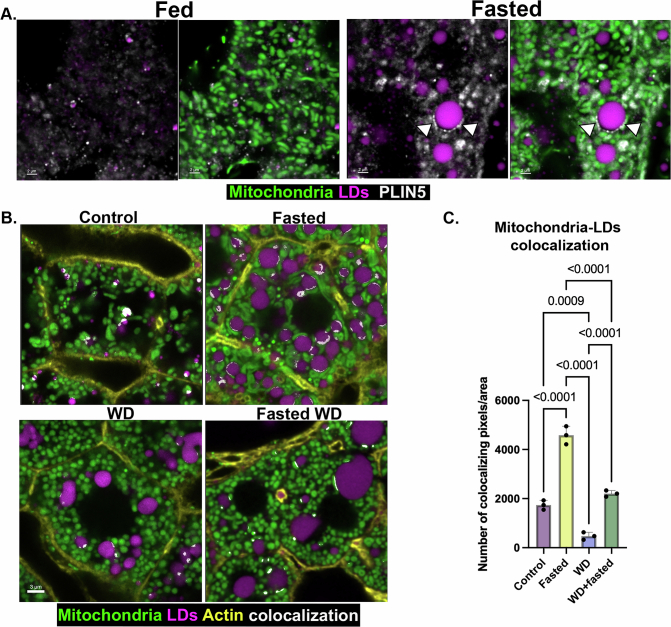
Fasting induces mitochondria-LD interactions. **(A)** Localization of endogenous PLIN5 in liver sections from fed and fasted Dendra2 mice. Scale bar 2 μm. **(B)** Confocal images of liver sections from mtDendra2 (green) transgenic mice fed control, WD, or overnight fasted or WD-fed-overnight fasted. LDs were labeled with Lipidtox (magenta) and actin with phalloidin (yellow). Colocalization between mitochondria and LD is shown in white. Scale bar 3 μm. **(C)** Mitochondria-LD colocalization was quantified as the overlapping pixels normalized to the area under different dietary conditions. Data presented as mean ± SD from n = 5 independent experiments. Statistical significance was assessed using one-way ANOVA with a Bonferroni correction for multiple comparisons. WD: western diet; LD: lipid droplets.

To understand how mitochondria–LD contacts form in the fasted liver, we examined the expression of proteins involved in mediating these interactions. In the fed liver, PP–PC zonation observed in the expression of proteins related to mitochondrial dynamics and LD formation (Fig. 4d,e, compare PP and PC columns in fed mice) might be related to the differences in mitochondrial topology and LD content (Fig. 1f,g). For instance, proteins associated with mitochondrial biogenesis were highly expressed in PP hepatocytes, consistent with our previous findings of greater mitochondrial mass in PP regions^17^. In response to fasting, many of these proteins were upregulated, aligning with the extensive remodeling observed using scPhenomics (Figs. 2 and 3). Notably, the LD coat protein PLIN5 was upregulated in the fasted liver (Fig. 4e). Indeed, basal PLIN5 levels in livers from CNTR and WD-fed mice were comparable, but doubled in response to fasting (Fig. 4f). Additionally, endogenous PLIN5 localized to the mitochondria–LD interface in fasted mice (Extended Data Fig. 5a). In summary, in homeostasis (that is, fed mice), conflicting metabolic processes are spatially separated at the protein level. However, in fasting, only the essential metabolic processes are upregulated and carried out by all hepatocytes.

To determine whether mitochondria–LD interactions can be remodelled in mice fed with WD, we fasted (overnight) WD-fed mice. These interactions were quantified using colocalization analysis in Imaris software (Extended Data Fig. 5b,c). Fasting induced mitochondrial elongation and enhanced mitochondria–LD interactions in both the fasted and WD-fasted mice. Notably, LD size increased, with larger droplets observed in fasted WD-fed mice, suggesting that mitochondria–LD contact sites might facilitate the channeling of lipids for storage in LDs (Extended Data Fig. 5b). Although 4 weeks of WD feeding causes steatosis, organelle dynamics remain functional and responsive to fasting, making it a valuable experimental model for manipulation.

### Mitochondria–LD contact sites promote LD expansion in fed mice

**Fig. 5:**
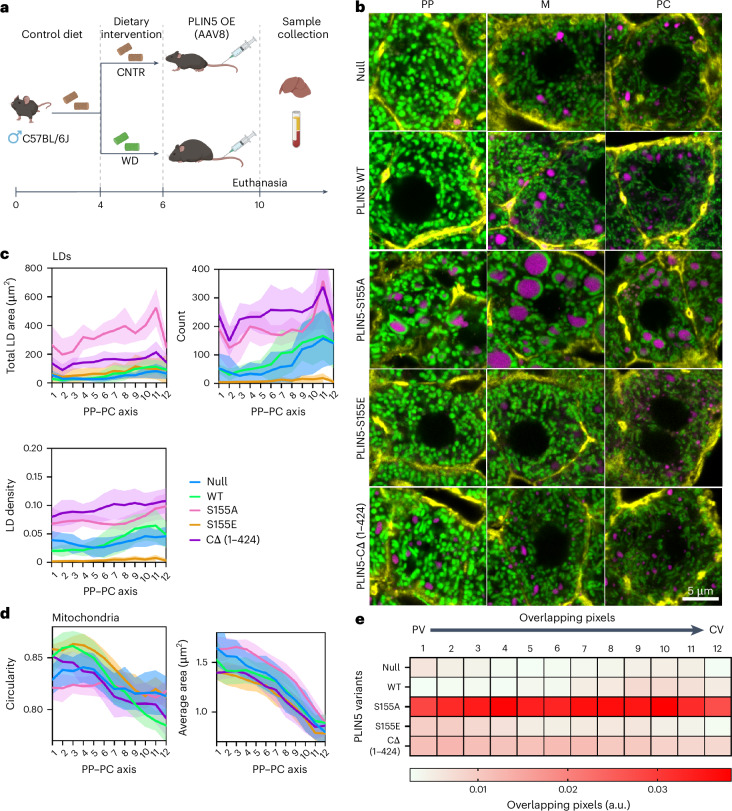
Mitochondria–LD contacts promote LD expansion. **a**, Experimental design schematic. **b**, Representative confocal images of periportal (PP), mid-lobular (M) and pericentral (PC) hepatocytes from mtDendra2 (green) mice fed CNTR and overexpressing PLIN5 variants. Actin is labelled with phalloidin (yellow), and LDs are labelled with LipidTox (magenta). **c**, scPhenomics of LD features, including total area, density and count, across the PP–PC axis from mice overexpressing PLIN5 variants. **d**, scPhenomics of mitochondrial features, including area and circularity, across the PP–PC axis from mice overexpressing PLIN5 variants. scPhenomics data are presented as mean ± s.d. from 12 PP–PC axes analysed from two mice. **e**, Heat map of mitochondria and LDs overlapping pixels across the PP–PC axis (R1–R12) from mice overexpressing PLIN5 variants. Icons in **a** created in BioRender; Porat-Shliom, N. https://BioRender.com/mzkgcrm (2026). Source data

To investigate the functional role of mitochondria–LD contact sites, we overexpressed PLIN5 in hepatocyes, using an AAV8 viral vector. Age-matched mice were fed either CNTR or WD for 2 weeks, followed by the overexpression of PLIN5 variants. Specifically, these included the WT PLIN5, a truncated version lacking the mitochondria-binding domain (PLIN5 CΔ (1–424)), and two phosphorylation mutants: a phospho-null (PLIN5-S155A) and a phospho-mimetic (PLIN5-S155E). Four weeks after viral transduction, liver tissue and serum samples were collected for microscopy and biochemical analysis (Fig. 5a and Supplementary Fig. 1).

**Extended Data Fig. 6:**
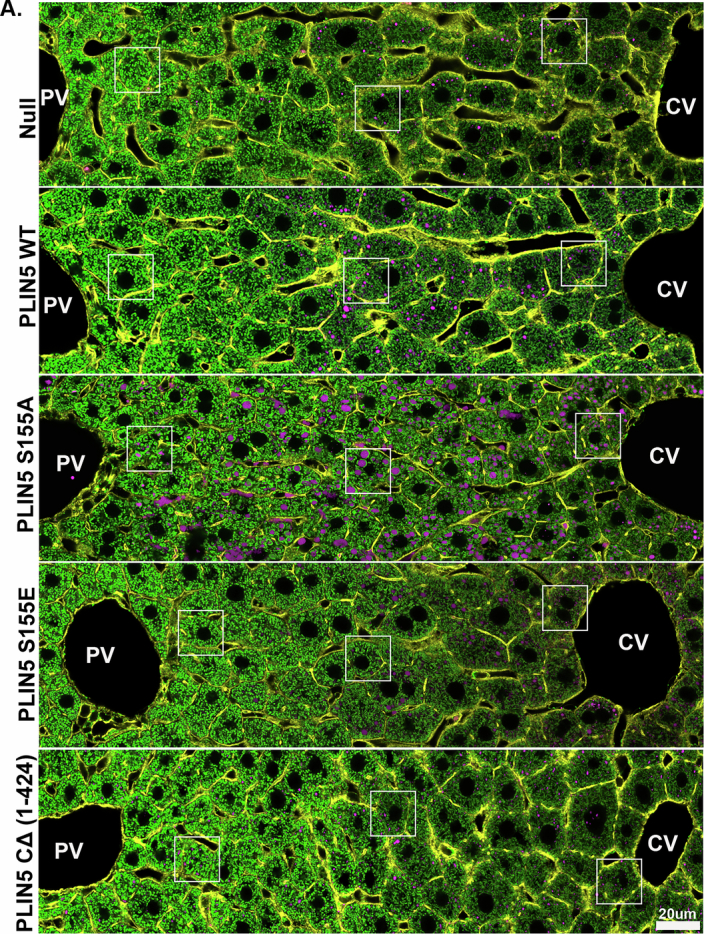
*In vivo* remodeling of mitochondria-LDs interactions using PLIN5 variants overexpression. **(A)** Representative confocal images of liver sections from control (CNTR) diet-fed mice overexpressing PLIN5 variants. Three mice per PLIN5 variant were analyzed. Images show mitochondria (green), LDs (magenta), and actin (yellow). Scale bar 20 μm. PV: portal vein; CV: central vein. WT: wild type.

Next, we assessed the impact of PLIN5 overexpression on LDs and mitochondria morphologies using scPhenomics. Representative images of PP, mid-lobular (M) and PC hepatocytes from mice fed a CNTR diet are shown in Fig. 5b (corresponding whole lobule images in Extended Data Fig. 6). When compared with the null, the overexpression of WT PLIN5 caused only minor changes in LD size, without changing their overall lobular distribution. However, the overexpression of WT PLIN5 had a considerbale effect on mitochondrial morphology. Hepatocytes 1–6 showed an increased circularity index (spherical morphology), whereas hepatocytes 7–12 exhibited a lower circularity index (tubular; Fig. 5d, comparing WT PLIN5 with the control null). Hepatocytes overexpressing PLIN5-S155A displayed elongated mitochondria closely associated with large LDs throughout the lobule, despite being fed the CNTR diet. By contrast, hepatocytes expressing PLIN5-S155E had fewer, smaller and more sparsely distributed LDs (Fig. 5c). Mitochondria in these cells were predominantly spherical and had a reduced mitochondrial area (Fig. 5d). Overexpression of the truncated PLIN5 CΔ (1–424) variant, which lacks the mitochondria-binding domain, did not affect mitochondrial morphology but led to the formation of small LDs throughout the lobule, likely owing to a dominant negative effect on lipolysis^29^. Finally, when comparing the ability of PLIN5 variants to promote mitochondria–LD interactions, PLIN5-S155A demonstrated the most pronounced effect across the entire lobule (Fig. 5e). Together, these findings suggest that PLIN5-mediated mitochondria–LD contact sites promote lipid storage in a phosphorylation-dependent manner.

### Mitochondria–LD contacts mitigate oxidative stress induced by 4-week feeding of WD

**Fig. 6:**
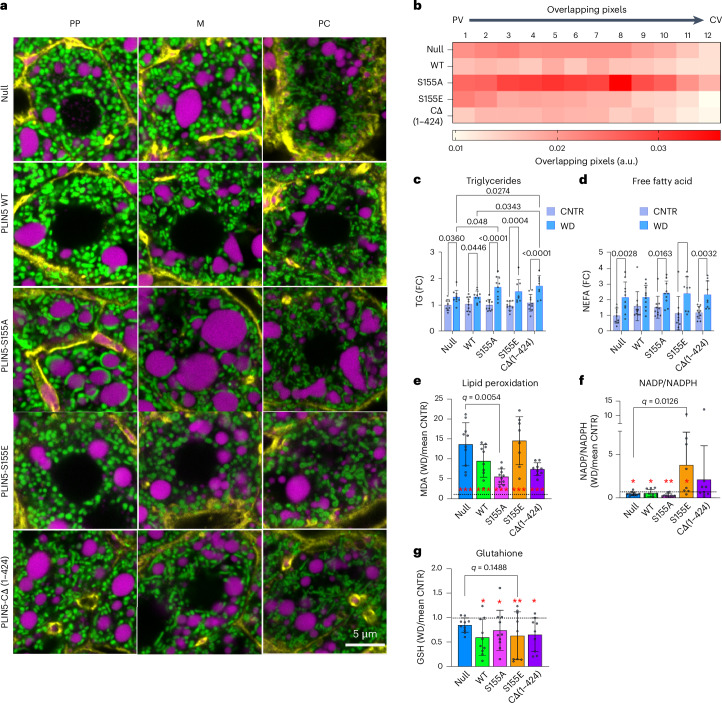
Mitochondria–LD contacts promote lipid storage, reducing WD-induced lipotoxicity. **a**, Representative confocal images of periportal (PP), mid-lobular (M) and pericentral (PC) hepatocytes from mtDendra2 (green) mice fed a WD and overexpressing PLIN5 variants. Actin is labelled with phalloidin (yellow), and LDs are labelled with LipidTox (magenta). **b**, Heat map of mitochondria and LDs overlapping pixels across the PP–PC axis (R1–R12) from WD-fed mice overexpressing PLIN5 variants. **c**,**d**, Triglyceride (**c**) and FFAs (**d**) were measured in the livers of mice overexpressing PLIN5 variants, fed either CNTR or WD. Statistical significance was calculated with two-way ANOVA and Tukey’s test to correct for multiple comparisons. Data presented as mean ± s.d. from *n* = 10 independent experiments. **e**–**g**, Malondialdehyde (MDA) (**e**), NADP/NADPH (**f**) and reduced glutathione (GSH) (**g**) were measured in the livers of mice overexpressing PLIN5 variants, fed either CNTR or WD. The dotted line represents WD equals mean CNTR. Values are presented as mean WD/CNTR diet ratios ± s.d. from *n* = 10 independent experiments. For each assay, a linear model was fit with diet (CNTR versus WD) and genotype as factors. The WD effect for each variant was compared to the Null group. In each variant group, WD was compared with the mean CNTR diet to test whether it differed from 1 (*q < 0.15; **q< 0.01; ***q < 0.01). Moderated *t*-statistics and associated *P* values were obtained using empirical Bayes shrinkage (eBayes). A multiple testing correction was applied across assays using the Benjamini–Hochberg method, and adjusted *P* values (*q* values) were calculated using two-way ANOVA. Source data

**Extended Data Fig. 7:**
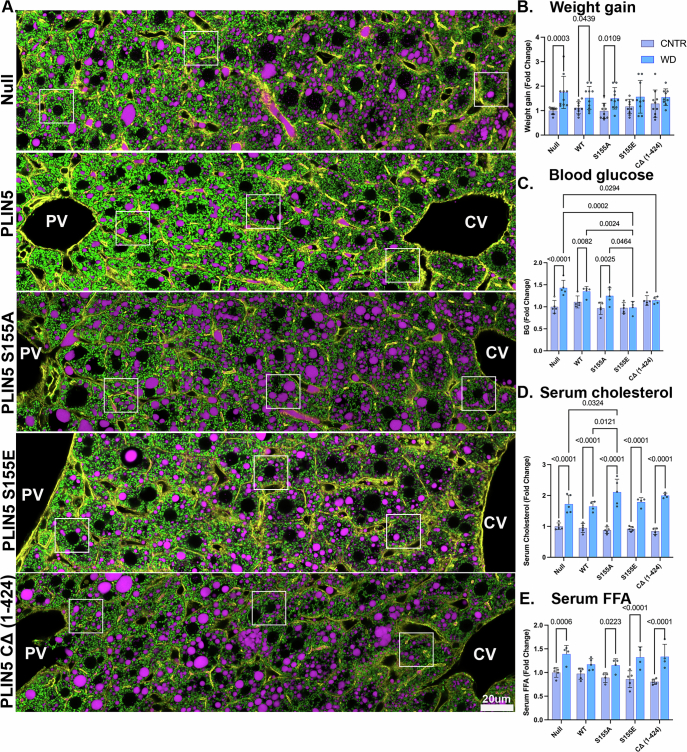
Overexpression of PLIN5 variants in WD-fed mice. **(A)** Representative confocal images of liver sections from Western Diet (WD)-fed mice overexpressing PLIN5 variants. Three mice per PLIN5 variant were analyzed. Images show mitochondria (green), LDs (magenta), and actin (yellow). Scale bar 20 μm. **(B)** Weight gain in mice overexpressing PLIN5 variants and fed CNTR or WD (n = 10 mice per diet). Serum FA **(C)**, cholesterol **(D)**, and glucose **(E)** levels in mice overexpressing PLIN5 variants and fed CNTR or WD (n = 5 per diet). Data presented as mean ± SD, statistical significance was calculated with two-way ANOVA and Tukey’s test to correct for multiple comparisons. PV: portal vein; CV: central vein; WT: wild type. CNTR: control; WD: western diet; FA: fatty acid. Source data

We examined the influence of PLIN5 overexpression on mitochondria–LD interactions in WD-fed mice. Representative images from PP, M, and PC hepatocytes are shown in Fig. 6a (corresponding whole lobule images, Extended Data Fig. 7a). Overexpression of WT PLIN5 in WD-fed mice noticeably affected mitochondria length, similar to that observed in the CNTR diet-fed mice. However, under these conditions, mitochondria–LD contact sites appeared more frequently than they did in CNTR diet-fed mice (Fig. 6a, comparing WT and null). In hepatocytes expressing PLIN5-S155A, mitochondria–LD contacts were observed throughout the lobule. By contrast, hepatocytes expressing PLIN5-S155E displayed mitochondria that were round and that rarely wrapped around LDs (Fig. 6a). In addition, the LD content in tissues expressing WT PLIN5 and PLIN5-S155E was lower than that in tissues expressing PLIN5-S155A (Extended Data Fig. 7a). As expected, the truncated variant, PLIN5 CΔ (1–424), failed to recruit mitochondria to LDs. Together, the phenotypes induced by overexpression of PLIN5 variants in the CNTR diet were consistent with those observed in WD. Furthermore, in both CNTR and WD mice, PLIN5-S155A and PLIN5-S155E variants impacted mitochondrial morphology and facilitated the assembly and disassembly, respectively, of mitochondria–LD contacts (Fig. 6a,b).

Next, we examined PLIN5 variant overexpression in mice fed either CNTR or WD affected whole-body and liver physiology. WD-fed mice gained weight compared with those on the CNTR diet. PLIN5 variant overexpression had no additional effect on body weight, serum cholesterol levels or FFA levels (Extended Data Fig. 7b,d,e). However, the increase in serum glucose levels, caused by WD, was reduced in mice expressing the PLIN5-S155E variant (Extended Data Fig. 7c). WD-fed PLIN5-S155A-expressing mice exhibited higher tissue TG levels than WD-fed null controls, consistent with mitochondria–LD contacts promoting TG storage in LDs (Fig. 6c). By contrast, while FFA levels approached saturation across all variants under WD, PLIN5-S155E showed the largest CNTR-to-WD shift, consistent with its higher lipolytic activity (Fig. 6d). These results support the hypothesis that mitochondria–LD interactions facilitate lipid storage.

**Extended Data Fig. 8:**
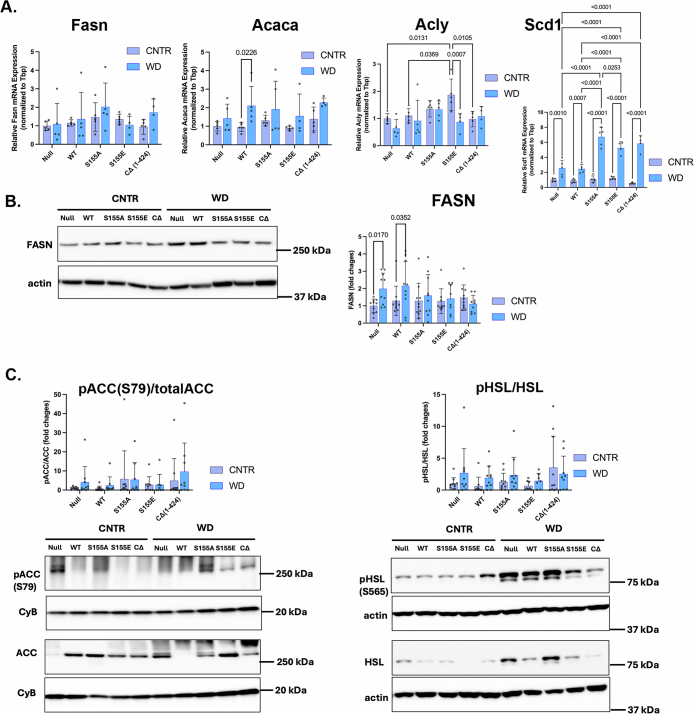
The effect of PLIN5 variants and WD on DNL. **(A)** Relative expression of mRNA levels of lipogenesis enzymes Fatty acid synthase (Fasn), Acetyl-CoA carboxylase 1 (Acaca), ATP citrate lyase (Acly), Stearoyl-CoA desaturase-1 (Scd1) in mice overexpressing PLIN5 variants fed a control diet (CNTR), or 4-week WD-fed. Data presented as mean ± SD from n = 5 independent experiments. Statistical significance was calculated using a two-way ANOVA and Tukey’s test to correct for multiple comparisons. **(B)** Fatty acid synthase (FASN) expression levels in mice overexpressing PLIN5 variants and fed CNTR or WD (n = 10 mice per diet). **(C)** Relative phosphorylated Acetyl-CoA carboxylase 1 (ACC1) and phosphorylated hormone-sensitive lipase (HSL) in mice overexpressing PLIN5 variants fed a control diet (CNTR), or 4-week WD-fed. Data presented as mean ± SD from n = 10 independent experiments. Statistical significance was calculated using a two-way ANOVA and Tukey’s test to correct for multiple comparisons. Representative Western blots are shown. DNL: de novo lipid synthesis; PV: portal vein; CV: central vein; WT: wild type. CNTR: control; WD: western diet. Source data

TG accumulation in PLIN5-S155A variant likely resulted from esterification of dietary fatty acids into LDs rather than increased de novo lipogenesis, as lipogenic enzyme abundance (RNA and protein) and phosphorylation of key regulatory enzymes were unchanged (Extended Data Fig. 8). WD and the overexpression of several PLIN5 variants and WD feeding had an additive effect on *Scd1* levels (Extended Data Fig. 8a). *Scd1* converts saturated FAs into monounsaturated FAs (MUFAs), a crucial step in the synthesis and storage of TGs in LDs^24,25^. MUFAs can also cooperate with PLIN5 to regulate oxidative gene expression^15,30^. Despite similar *Scd1* upregulation across variants, only PLIN5-S155A variant displayed mitochondria–LD contacts sites and showed larger LDs and higher Tg content, supporting a model in which mitochondria–LD interactions preferentially channel excess lipids into TG storage.

Excess FAs in the WD cause oxidative stress and damage to membranes, leading to hepatocyte injury and inflammation^31–33^. We hypothesized that mitochondria–LD interactions reduce lipotoxicity by promoting the storage of excess FFAs. To test this, we measured NADP/NADPH levels, reduced glutathione (GSH) and malondialdehyde (MDA). The measured values were log_2_-transformed and analysed in R using the limma package. For each assay, a linear model was fit with diet (CNTR versus WD) and PLIN5 variant as factors. Two contrasts were tested: (1) assessing the influence of the WD with each variant by comparing WD samples to the mean of the CNTR diet (that is, whether the linear WD/CNTR fold change differs from 1, and (2) assessing whether the WD effect in each variant differed from that in the null group. This comparison whether the PLIN5 variant’s WD/CNTR ratio differs from the null’s WD/CNTR ratio.

Four weeks of WD feeding led to increased MDA, a reporter for lipid peroxidation, in null control mice (Fig. 6e). Although the MDA levels were higher in all WD-fed mice overexpressing a PLIN5 variant, only PLIN5-S155A levels were reduced compared to the null control, suggesting that mitochondria–LD contacts mitigate lipid peroxidation (Fig. 6e). The opposite trend was measured with the redox state indicator NADP/NADPH. Four weeks of WD feeding resulted in lower NADP/NADPH levels in null, WT and PLIN5-S155A mice. However, the PLIN5-S155E group had higher NADP/NADPH levels than did the null group, indicating an oxidative, potentially damaging environment (Fig. 6f). Last, reduced glutathione serves as an antioxidant, alleviating oxidative stress. Levels of GSH were lower in PLIN5-S155E mice, consistent with a more oxidizing and potentially harmful environment (Fig. 6g). Collectively, by sequestering excess FA into LDs, mitochondria–LD interactions mitigate cellular damage caused by the overabundance of FFAs.

### Mitochondria–LD contacts are associated with higher lipid content in mice and humans

**Extended Data Fig. 9:**
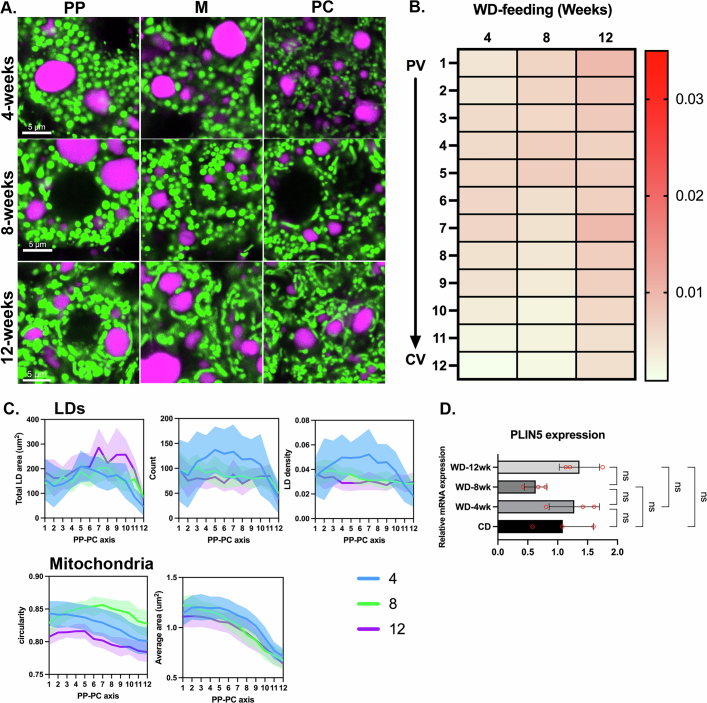
Increased mitochondria-LD interactions correlate with prolonged WD-feeding in mice. **(A)** Representative confocal images of liver sections from mtDendra2 mice fed WD for 4, 8, and 12 weeks. Mitochondria (green), LDs (magenta), and actin (yellow). Scale bar 5 μm. **(B)** Heat map of mitochondria and LDs overlapping pixels across the PP-PC axis (R1-R12) and prolonged WD feeding. **(C)** Relative *Plin5* mRNA expression in mice fed WD for 4,8, and 12 weeks. **(D)** Relative PLIN5 mRNA expression in mice fed a control (CNTR) diet or Western Diet (WD) for 4, 8, and 12 weeks. Data presented as mean ± SD from n = 3 independent experiments. Statistical significance was calculated using a two-way ANOVA and Tukey’s test to correct for multiple comparisons. PV: portal vein; CV: central vein. WT: wild type. Source data

To investigate whether mitochondria–LD interactions are associated with lipid buildup over time, mtDentra2 mice were fed a WD for 4, 8 or 12 weeks. Liver sections were labelled, imaged and analysed with scPhenomics. Mitochondria–LD contacts were more frequent after 12 weeks on WD (Extended Data Fig. 9a,b). Following 12 weeks of WD feeding, LD total area increased, and mitochondria became tubular, indicated by lower circularity values (Extended Data Fig. 9c). The mRNA expression levels of *Plin5*, however, were unchanged (Extended Data Fig. 9d).

**Fig. 7:**
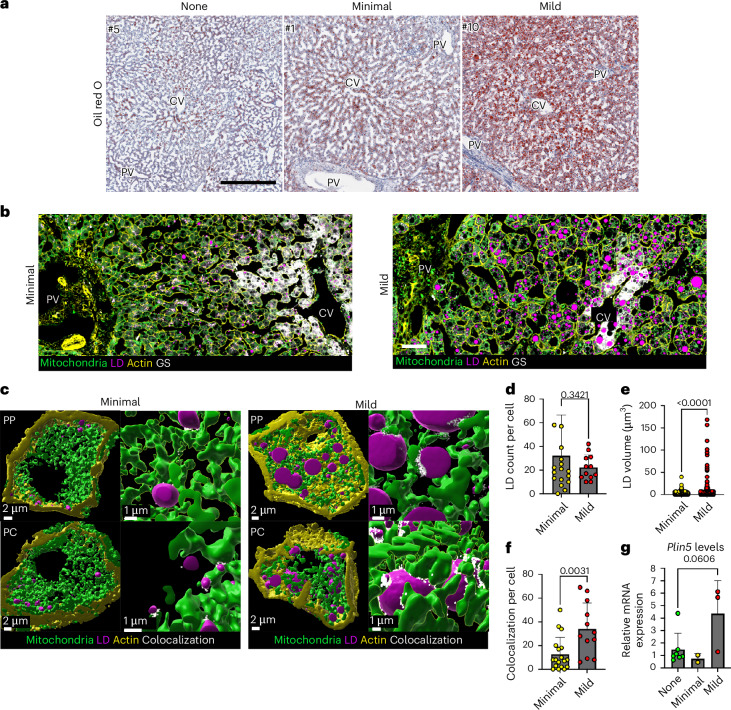
Mitochondria–LD contacts are associated with higher lipid content in the human liver. **a**, Histopathology was used to group 12 healthy samples on the basis of lipid content. Oil red O staining of a representative individual from each group. Scale bar, 500 μm. **b**, Immunofluorescence staining of mitochondria (green), LDs (magenta), actin (yellow) and GS (white) in a liver section from a representative individual with minimal or mild steatosis. Scale bar, 100 μm. **c**, Volume rendering of mitochondria (green), LDs (magenta) and actin (yellow) in a representative PP and PC hepatocytes from patients with minimal or mild steatosis. Colocalizing pixels are shown in white. **d**–**f**, Quantification of LD count (**d**), LD volume (**e**) and mitochondria–LD colocalization (**f**), in *n* = 16 cells from 2 people with minimal steatosis and *n* = 12 cells from 3 people with mild steatosis, presented as mean ± s.d. Statistical significance was calculated using a one-way ANOVA. **g**, *Plin5* expression was assessed in liver samples from seven people without steatosis, two with minimal steatosis and three with mild steatosis. Data presented as mean ± s.d. Statistical significance was calculated using a one-way ANOVA. Statistical significance was calculated using a *t*-test. Source data

The association between mitochondria–LD contacts was also examined in the human liver. Histopathology was performed on 12 liver biopsies from healthy donors (10 females and 2 males; donor characteristics are described in Methods). Steatosis in the samples is incidental, of unknown origin, and reflects the prevalence of steatosis in the population. The samples were divided into three groups based on lipid content (Fig. 7a and Supplementary Table 1). To examine mitochondria–LD interactions, liver sections were stained to label mitochondria and LDs (Fig. 7b). High-resolution *z*-stacks of PP and PC hepatocytes were captured, and cellular and organelle surfaces were rendered using Imaris software (Fig. 7c). Although there was no difference in the number of LDs per cell (Fig. 7d), the LD’s volume distribution in the ‘mild’ group was notably higher (Fig. 7e), as was the extent of mitochondria–LD colocalization per cell (Fig. 7f). Despite the limited sample size, *Plin5* mRNA levels were higher in samples displaying higher lipid content, consistent with mitochondria–LD interactions (Fig. 7g). These findings establish a correlation between lipid content and mitochondrial–LD interactions, as well as their remodeling during chronic WD exposure.

**Fig. 8:**
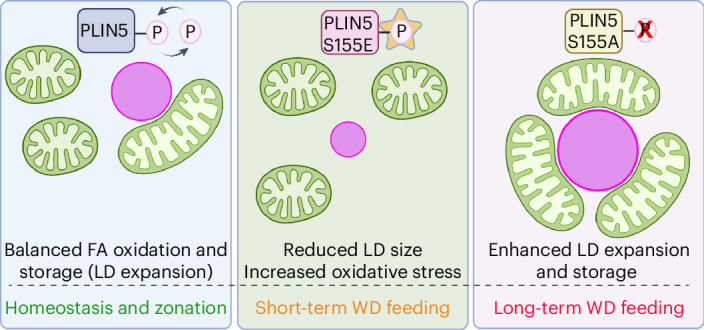
PLIN5-induced mitochondria-LD contacts regulate lipid homeostasis. Proposed model of mitochondria–LD contacts regulation by PLIN5. PLIN5 induces mitochondria–LD contacts in a phosphorylation-dependent manner. PLIN5-S155A induces contact sites promoting the esterification of FFA to form TG and storage in LDs, which is protective from oxidative stress owing to increased dietary lipids. In PLIN5-S155E, mitochondria and LDs are rarely associated; LDs are smaller, and antioxidant capacity is reduced. In long-term WD feeding, the abundance of mitochondria–LD contacts increases, suggesting that the assembly of these contacts might be part of an adaptive response. Created in BioRender; Porat-Shliom, N. https://BioRender.com/jnbti4d (2026).

In summary, we propose that phosphorylation of PLIN5 controls mitochondria–LD contacts and their function in maintaining lipid homeostasis (Fig. 8). The phospho-null PLIN5-S155A increases these contacts, promoting free-fatty-acid esterification into triglycerides for LD storage and protecting against oxidative stress, whereas the phospho-mimetic PLIN5-S155E vaiant reduces contacts, yields smaller LDs and lowers antioxidant capacity. During long-term WD feeding, mitochondria–LD contacts become more abundant, indicating that these contact sites can become harmful owing to prolonged lipid exposure.

## Discussion

This study examined how increased lipid flux affects the structure, organization and function of organelles across different liver zones. We developed a single-cell-resolution imaging-based technique to interrogate organelle topology in the native tissue environment. Applying scPhenomics to map changes in organelles, we identified distinct zonal patterns that depend on nutritional status. To our knowledge, using organelle features as classifiers of function has mainly been applied to cells in culture. This phenotypic profiling offers insights into liver zonation and organelle interactions, highlighting their functional roles in tissue adaptation to metabolic cues. Our study also highlights the utility of scPhenomics in identifying spatially relevant phenotypes that can be integrated with other single-cell methods^18,34,35^.

One prominent structural adaptation was the remodelling of mitochondria–LD contact sites in response to nutritional stress. In fasted livers, mitochondria–LD contacts spanned the lobule, whereas in WD-fed tissues, they were scarce (Figs. 2b,c and 3e). These differences link mitochondrial–LD interactions with lipid metabolism, consistent with prior studies linking them with lipid oxidation or storage^36–39^. We show that mitochondria–LD contacts facilitate FA esterification to form TG for storage, thereby reducing lipotoxicity by confining potentially harmful FFA in LDs. This explains the high abundance of mitochondria–LD interactions in the fasted liver (Fig. 2b). The absence of mitochondria–LD interactions in short-term WD-fed mice is consistent with inefficient storage of FFA in LDs, which contribute to inflammation and cell damage. To examine this mechanistically, we identified proteins involved in mitochondria–LD interactions using spatial proteomics.

The LD coat protein PLIN5 was upregulated in the fasted liver. On the surface of LDs, PLIN5 inhibits lipolysis by directly binding to adipose triglyceride lipase, thus indirectly promoting LD expansion^29^. During fasting, however, protein kinase A phosphorylates PLIN5 on S155, facilitating the release of FAs through lipolysis^15,30,37^. We found that in vivo overexpression of PLIN5-S155A promoted extensive mitochondrial–LD interactions, increased TG content and enlarged LD size. Conversely, mitochondria did not interact with LD in PLIN5-S155E-overexpressing livers, consistent with the dissociation of PLIN5 and the utilization of FAs. Interestingly, in starved myoblasts, PLIN5 phosphorylation promotes FA transport into the mitochondria for oxidation, rather than storage in LDs^38^. These findings suggest that PLIN5-mediated mitochondrial–LD contacts direct lipid flux differently across cell types and nutritional states. We were unable to measure the direction of FA transfer in the current study owing to the challenges of performing this assay in vivo. Further experiments are needed to fully elucidate the regulatory mechanisms across organ systems and nutritional states.

Several studies have reported that fasting induces the formation of the mitochondria–LD contact site^2,3^. Interestingly, mitochondria–LD contact sites have also been reported in mice fed a high-fat diet and in genetic mouse models of obesity^16,40^. Although we detected mitochondria–LD contacts in fasted mice, we did not initially observe them in short-term WD-fed mice. Extending WD feeding in mice was accompanied by a rise in mitochondria–LD interactions (Extended Data Fig. 9). In the healthy human liver, higher mitochondria–LD colocalization was observed in livers with higher lipid content, which was also associated with higher PLIN5 expression (Fig. 7d–g). Overall, our results show that mitochondria–LD contacts are remodelled over time, consistent with the progressive nature of steatotic liver disease^41,42^

During short-term WD exposure, mitochondria–LD contacts protect from oxidative stress. Overexpression of PLIN5 S155A reduced lipid peroxidation and improved redox imbalance, while PLIN5 S155E worsens these effects (Fig. 6e–g). Increasing PLIN5 benefits lipid and glucose regulation, promoting cellular health^14,43,44^. We propose that fasting-related lipid influx remodels mitochondria–LD contacts to support storage and prevent lipotoxicity, whereas in nutrient excess, this adaptive response is disrupted, leading to harmful lipid buildup. Notably, PLIN5-S155A overexpression in WD-fed mice reduces damage by channeling FFA into LDs. However, prolonged WD exposure could turn mitochondria–LD contacts into contributors to disease progression (Fig. 7 and Extended Fig. 9). Because FFA levels are not reduced by PLIN5 overexpression, PLIN5 overexpression could protect against lipotoxicity through an alternative mechanism. Furthermore, our study supports the notion that PLIN5-mediated contacts promote FFA esterification; however, it remains inconclusive whether mitochondria attached to LDs contribute to DNL. Further experiments are needed to determine whether dissociation of mitochondrial–LD interactions can reverse and prevent disease progression. Supporting this is a recent study showing PLIN5 deletion protects against metabolic dysfunction-associated steatotic liver disease^45^. Overall, these findings highlight the critical role of dynamic contact sites in lipid management and cellular health (Fig. 8).

Lipid-related pathways are compartmentalized across different organelles^16,46,47^. Fasting led to the upregulation of proteins involved in membrane contact sites, indicating enhanced organelle interactions (Fig. 4e). This suggests rewiring of organelle–organelle interactions beyond mitochondria and LDs^5,37^. Notably, Mitofusin 2 (Mfn2) and Mitoguardin 2 (MIGA2), both of which localize to the ER, facilitate lipid transfer between mitochondria and the ER, and subsequently store lipids in LDs^48,49^. Because the ER plays a key role in lipid metabolism and glycaemic control, the benefits of PLIN5-S155A overexpression in high-fat diet mice likely involve ER-mediated processes. Future studies involving scPhenomics could deepen understanding of these organelle interactions^36,50,51^.

The study underscores the critical role of mitochondrial morphology and organelle interactions in metabolic flexibility. In the fed state, mitochondrial subpopulations facilitate lipid oxidation and storage in PP and PC, respectively^37,39,52^. Nutritional stress, such as fasting, triggers a unified mitochondrial response across the liver, driven by PLIN5 modulation, enabling simultaneous lipid oxidation to support ketogenesis and temporary storage in LD to avoid damage. These dynamic interactions are vital for metabolic adaptation and might be disrupted in chronic liver disease, influencing liver cancer development^35,53,54^.

## Methods

### Animal experiments

Experiments were approved by the Institutional Animal Care and Use Committee of the National Cancer Institute and in compliance with the Guide for the Care and Use of Laboratory Animals (National Institutes of Health publication 86-23, revised 1985). Mice were housed in a temperature-controlled facility (21–24 °C) under a 12 h light/12 h dark cycle (lights on/off at 06:00/18:00). Because ambient temperature and circadian phase strongly influence whole-body metabolism, all metabolic experiments and tissue-collection procedures were performed at matched zeitgeber times, with mice acclimated to housing conditions for at least 7 days before phenotyping. All experiments were conducted on mice fed ad libitum normal chow diet (NIH-31 Open Formula) or WD (TD.120528, Envigo), aged 4–10 weeks C57BL/6J (strain no. 000664) or mtDendra2 excised^19^ (photo-activatable mitochondria; strain no. 018397) male mice obtained from Jackson Laboratories.

For PLIN5 overexpression, 4-week-old C57BL/6J male mice and mtDendra2 excised male mice were housed in a temperature-controlled facility (21–24 °C) under a 12 h light/12 h dark cycle (lights on at 07:00). Mice were fed either a control chow or WD for 2 weeks. They were then injected through the tail vein with an AAV8-ABLp vector—Null, mPlin5 WT, S155A, S155E or CΔ (1-424)—driven by a liver-specific albumin promoter, at a titer of 4.5 × 10^11^ GC (Vector Biolabs). After AAV8 transduction, mice were fed the control chow diet or WD for 4 weeks. At the end of the experiment, serum and liver tissue were collected.

### Intra-cardiac fixation, tissue processing and immunofluorescence

Mice were anaesthetized with 250 mg kg^−1^ body weight xylazine and 50 mg kg^−1^ body weight ketamine (diluted in saline), injected intraperitoneally (i.p.). The liver was fixed by transcardial perfusion of ice-cold PBS for 2 min followed by ice-cold 4% paraformaldehyde (PFA) in PBS at a rate of 5 ml min^−1^. Livers were collected and stored in 4% PFA in PBS overnight and processed in a sucrose gradient before they were embedded in OCT (Tissue-Tek). Blocks were kept at −80 °C, until 10-µm-thick slices were made with a cryostat and slides were prepared. Slides were stored at −80 °C until thawed, rehydrated and blocked with 0.1% Triton X-100 and 10% FBS in PBS for 1 h at room temperature. Next, slides were incubated with primary antibody at 4 °C overnight. The following day, the slides were washed three times for 15 min each, and then incubated with a secondary antibody for 1 h at room temperature. After three 15 min washes, slides were mounted with Fluoromount-G and a coverslip (no. 1).

### Confocal microscopy and scPhenomics

Tile scans and *z* stacks were acquired using a Leica SP8 inverted confocal laser scanning microscope using a ×63 oil objective and a 1.4 numerical aperture.

Whole-lobule images were analysed using a custom Python script (Python v. 3.10) using the deep-learning Cellpose package for segmentation^20^. Individual hepatocytes were identified by first creating a combination channel of actin and Gaussian-blurred mitochondria. We applied a custom-trained Cellpose model to this combination channel to generate hepatocyte segments. The central and portal veins were manually segmented, and their total length was measured. Segmented hepatocyte distance to the central vein was normalized to the total PP–PC length and then binned into 12 regions to ensure at least one hepatocyte per bin.

Mitochondria and LDs were segmented using their own custom-trained Cellpose models. LD segments were also subjected to an intensity threshold to remove false-positive segments. The mitochondria and LD segments were used to quantify organelle counts per cell, densities, mitochondrial and lipid fluorescence intensities and geometric parameters, including total and average area, average perimeter, circularity, eccentricity, solidity and feret diameter using the region props function from scikit-image^55^. The shortest mitochondria to lipid droplet distance, percent mitochondria/lipid droplet overlap, and mitochondria Circularity = 4 × pi × area / perimeter^2^ were quantified. Investigators were blinded to group allocation during data collection and analysis.

PCA was performed using FactoMineR version 2.11R package. The following variables were used in calculations: counts, total area, average area, average perimeter, average eccentricity, average solidity, average feret, average mito int, average lipid int, average organelle min dist, average circularity and average density. Unsupervised clustering was performed using the hclust (method = ‘complete’) command of the stats version 4.3.0R package, and heatmaps were visualized using pheatmap version 1.0.12. Correlation values were calculated with default settings (‘cor’ command of ‘stats’ R package), rounded to two decimal places, and visualized using the ggplot function.

For 3D evaluation, surfaces were generated using Imaris (Bitplane 9.9.0) for mitochondria, LDs and the whole cell. Geometrical measurements were exported into Excel.

### Histology

Mouse liver tissues were also used for independent histological analysis of liver steatosis with hematoxylin and eosin by Histoserv.

### Liver perfusion and hepatocyte isolation

Livers of anaesthetized mice were perfused by inserting a 22-gauge syringe into the portal vein and delivering 25 ml of pre-warmed (37 °C) perfusion buffer (Krebs-Henseleit buffer (Sigma) with 0.5 μM EDTA), followed by 50 ml collagenase A buffer (Krebs-Henseleit buffer, with 0.1 mM Ca2Cl and 0.4 mg ml^−1^ collagenase A (Sigma)). After perfusion, livers were transferred into a Petri dish and flooded with cold PBS, and hepatocytes were gently released using forceps. Dissociated cells were collected and filtered through a 100-μm cell strainer. Cells were centrifuged at 100*g* for 5 min at 4 °C to obtain a hepatocyte enriched pellet. Pellets were resuspended in cold PBS, filtered through a 40-μm cell strainer and centrifuged at 100*g* for 5 min at 4 °C. Percoll diluted in 10× Hank’s buffer (Sigma) was added to cell suspension and centrifuged at 100*g* for 5 min at 4 °C. The supernatant containing dead cells was removed by aspiration. The pellet was resuspended in cold PBS, and the number of cells was determined.

### Fluorescence-activated cell sorting of PP and PC populations

Cell sorting was performed on a FACSAria Fusion cell sorter (BD Biosciences). Forward and side light scatter was used to distinguish cells from debris and to identify single cells. Zombie-Green fixable live dead dye (1:500, BioLegend) was used to discriminate live and dead cells. The PP and PC populations were identified using anti-E-cadherin-PE (1:100; clone: DECMA-1; cat. no. 147306; BioLegend) and anti-CD73-APC antibody staining (1:150; clone: TY/11.8; cat. no. 127210; BioLegend). The PP- and PC-specific gates were set after spectral compensation, using appropriate fluorescent-minus (FMO) controls.

### Protein digestion and TMT labeling

Cells were lysed in 50 mM HEPES, pH 8.0, 8 M urea and 10% methanol, followed by sonication. Lysates were clarified by centrifugation, and the protein concentration was quantified using a BCA protein estimation kit (Thermo Fisher). A 250 µg was alkylated and digested by incubating overnight at 37 °C in trypsin at a ratio of 1:50 (Promega). Digestion was acidified by adding formic acid (FA) to a final concentration of 1% and desalted using Pierce peptide desalting column, following the manufacturer’s protocol. Peptides were eluted from the column using 50% ACN and 0.1% FA, dried in a speedvac and kept frozen at −20 °C for further analysis.

For TMT labelling, 125 µg of each sample was reconstituted in 50 μl of 50 mM HEPES, pH 8.0, 500 µg of TMTpro-16plex label (Thermo Fisher) in 100% ACN. After incubating the mixture for 1 h at room temperature with occasional mixing, the reaction was terminated by the addition of 8 μl of 5% hydroxylamine. Because there were more than 16 samples, we generated a pooled sample consisting of equal amounts of lysate from each condition and TMT labelled. The pool was added to each TMT experiment. The TMT-labelled peptides were pooled and dried in a speedvac. The samples were desalted, and excess TMT label was removed using peptide desalting columns (Thermo Fisher). A 100-µg aliquot of labelled peptide mixture was fractionated using a high pH reversed phase.

### High pH reversed-phase fractionation

The first-dimensional separation of peptides was performed using a Waters Acquity UPLC system coupled with a fluorescence detector (Waters) using a 150 mm×3.0 mm Xbridge Peptide BEMTM 2.5 μm C18 column (Waters) operating at 0.35 ml min^−1^. The dried peptides were reconstituted in 100 μl of mobile phase A solvent (3 mM ammonium bicarbonate, pH 8.0). Mobile phase B was 100% acetonitrile (Thermo Fisher). The column was washed with mobile phase A for 10 min followed by gradient elution 0–50% B (10–60 min) and 50–75% B (60–70 min). Fractions were collected every minute. These 60 fractions were pooled into 24 fractions. Fractions were vacuum centrifuged, and lyophilized fractions were stored at −80 °C until analysis by mass spectrometry.

### Mass spectrometry acquisition

The lyophilized peptide fractions were reconstituted in 0.1% TFA and subjected to nanoflow liquid chromatography (Thermo Ultimate 3000RSLC nano LC system, Thermo Fisher) coupled to an Orbitrap Eclipse mass spectrometer (Thermo Fisher). Peptides were separated using a low pH gradient using a 5–50% ACN over 120 min in a mobile phase containing 0.1% formic acid at 300 nl min^−1^ flow rate. MS scans were performed in the Orbitrap analyser at a resolution of 120,000 with an ion accumulation target set at 4 × 10^5^ and max IT set at 50 ms over a mass range of 400–1,600 *m/z*. Ions with a determined charge state between 2 and 5 were selected for MS2 scans in the ion trap with CID fragmentation (Turbo; NCE 35%; maximum injection time 35 ms; AGC 1 × 10^4^). The spectra were searched using the Real Time Search Node in the tune file using mouse Uniprot database using Comet search algorithm with TMT16 plex (304.2071 Da) set as a static modification of lysine and the N termini of the peptide. Carbamidomethylation of cysteine residues (+57.0214 Da) was set as a static modification, whereas oxidation of methionine residues (+15.9949 Da) was set up as a dynamic modification. For the selected peptide, an SPS–MS3 scan was performed using up to 10 b- and y-type fragment ions as precursors in an Orbitrap at 50,000 resolution with a normalized AGC set at 500 followed by maximum injection time set as ‘Auto’ with a normalized collision energy setting of 65.

### Proteomics data analysis

Acquired MS/MS spectra were searched against the mouse Uniprot protein database along with a contaminant protein database using SEQUEST in the Proteome Discoverer 2.4 software (Thermo Fisher). The precursor ion tolerance was set at 10 ppm and the fragment ions tolerance was set at 0.02 Da, with methionine oxidation included as a dynamic modification. Carbamidomethylation of cysteine residues and TMTpro16 plex (304.2071 Da) were set as static modification of lysine and the N terminus of the peptide. Trypsin was specified as the proteolytic enzyme, with up to two missed cleavage sites allowed. Searches used a reverse sequence decoy strategy to control for the false peptide discovery and identifications were validated using the Percolator algorithm in Proteome Discoverer 2.4.

Reporter ion intensities were adjusted to correct for lot-specific impurities according to the manufacturer specification, and protein abundancies were quantified using the summation of the reporter ions for all identified peptides. A two-step normalization procedure was applied on a 24-plex TMT experiment (2 TMT experiments with 12 samples each). First, the reporter abundances were normalized across all the channels to account for equal peptide loading. Second, the intensity of the pooled sample was used to normalize the batch effect from the multiple TMT experiments. Samples were further normalized using the voom algorithms and quantile normalization from the Limma R package (v3.40.6). Differential peptide expression analysis was performed using Limma, where zonation was determined by the *P* (0.05) and/or FC (≥1.2); pathway enrichment analysis was performed using Fisher’s Exact test with the GO database. Graphpad Prism 9 and Biorender was used for visualization.

The mass spectrometry dataset has been deposited in the MassIVE database under accession code MSV000093282.

### Serum analysis

Kits were used, following to manufacturer’s instructions, to measure, free fatty acid FUJIFILM (Wako Diagnostics), triglycerides (Pointe Scientific) and cholesterol (Thermo Scientific).

### Blood glucose

Blood was collected from the tail vein, and glucose was measured using testing strips and a monitor (Ascencia Diabetes Care).

### Triglyceride, malondialdehyde and glutathione measurements

Pulverized liver tissue samples were lysed, and the supernatant was collected after centrifugation at 16,000*g* for 10 min. Triglyceride levels were measured with a Colorimetric Assay Kit (Elabscience), cellular malondialdehyde (MDA) levels were measured with a Lipid Peroxidation (MDA) Assay Kit (Abcam), and NADP/NADPH (Sigma MAK479) and total cellular glutathione (GSH) levels were estimated through the GSH Glo Assay Kit (Promega). Each kit was used according to the manufacturer’s instructions. Results were normalized to protein amount, and absolute numbers were converted to fold change.

### Western blot analysis

Proteins from primary hepatocytes or pulverized liver were extracted by homogenization in RIPA lysis buffer (150 mM NaCl, 0.1% Triton X-100, 0.5% sodium deoxycholate, 0.1% sodium dodecyl sulfate, and 50 mM Tris HCl pH 8.0) containing EDTA, PMSF and Halt Inhibitor Cocktail (Thermo Fisher), followed by centrifugation at 16,000*g* at 4 °C for 30 min. Protein concentration was determined using a Pierce BCA Protein Assay Kit (Thermo Fisher). Lysates were boiled at 95 °C for 5 min, and 5- to 10-μg aliquots were fractionated by SDS–PAGE and then transferred to a nitrocellulose 0.45-µm membrane (Bio-Rad). Membranes were blocked for 1 h at room temperature in 5% non-fat milk in 1× Tris-buffered saline + 0.1% Tween 20 (TBST) and then incubated with primary antibody diluted in 5% BSA in 1× TBST at 4 °C overnight. Membranes were washed three times with TBST and then incubated in the secondary antibody (HRP-linked anti-mouse IgG; Cell Signaling; cat. no. 3662S; 1:2,500 or anti-rabbit IgG, HRP-linked antibody; Cell Signaling; cat. no. 7076S; 1:10,000) diluted in 5% non-fat milk in 1× TBST for 1 h at room temperature. Membranes were washed three times with TBST, and then Clarity ECL Western blot substrate solution (Bio-Rad) was applied for detection. The membranes were imaged using the ChemiDoc Imaging System (Bio-Rad). Bands were quantified with ImageLab (Bio-Rad).

Primary antibodies used were: rabbit monoclonal antibody to cyclophilin B (D1V5J) from Cell Signaling (cat. no. 43603S; clone no. D1V5J), dilution 1:1,000. Antibody to phospho-HSL (Ser565) from Cell Signaling (cat. no. 4137S), dilution 1:1,000. Rabbit monoclonal antibody to β-actin (cat. no. 4970S; clone no. 13E5), dilution 1:1,000. Mouse monoclonal antibody to β-actin from Cell Signaling (cat. no. 3700S; clone no. 8H10D10), dilution 1:1,000. Rabbit monoclonal antibody to β-tubulin from Cell Signaling (cat. no. 2128S; clone no. 9F3), dilution 1:1,000. Polyclonal antibody to perilipin 5 from Proteintec (cat. no. 26051-1-AP), dilution 1:2,000. Polyclonal antibody to OXPAT from Invitrogen (cat. no. PA5-114352), dilution 1:500. Antibody to GAPDH from Cell Signaling (cat. no. 2118S; clone no. 14C10), dilution 1:1,000.

### RNA isolation and quantitative PCR

RNA was isolated from pulverized liver tissue using the TissueLyser LT and RNeasy mini kit (Qiagen). A High-Capacity RNA to cDNA kit (Thermo Fisher) was used to synthesize random-primed cDNA from 2 μg DNAse treated RNA. Real-time PCR was conducted in 384-well plates using a ViiA7 Real-Time PCR system (Applied Biosystems). Singleplex reactions (5 μl) containing a FAM-MGB expression assay for Plin5 (Mm00508854_m1) or Tbp control (Mm01277041_m1) (Thermo Fisher) were performed using cDNA synthesized from 8 ng RNA and 1× Fast Advanced Master Mix (Thermo-without Amp Erase UNG) were performed in triplicate. The comparative 2^−ΔΔCt^ method was used to determine relative expression normalized to Tbp (Applied Biosystems ViiA 7Real-Time PCR System Getting Started Guides).

### Human liver sample collection

Human tissue was acquired with written informed consent under an NIH IRB-approved protocol (NCT01915225) during risk-reducing surgery performed on people with a germline mutation in the tumour suppressor CDH1. Substitutions in CDH1 are a frequent cause of the hereditary diffuse gastric cancer (HDGC) syndrome, which is associated with gastric adenocarcinoma, lobular breast cancer and cleft lip and palate. People with this disease undergo risk-reducing total gastrectomy, and some consent to a liver biopsy during the surgery. Although these patients often have microscopic stage 1A cancers, none had deeply invasive or metastatic carcinoma in their livers.

The tissue procured was characterized as normal upon visible inspection, followed by histopathological evaluation. Tissue was procured within approximately 20 min of the incision and then fixed in 1% PFA in PBS for 48 or 72 h. It was then washed in PBS and incubated in 30% sucrose in PBS for at least 24 h before being embedded in OCT. OCT blocks were stored at −80 °C.

### Statistical analyses

No statistical methods were used to pre-determine sample sizes, but our sample sizes are similar to those reported in previous publications^7,52,56^. The data distribution was assumed to be normal, but this was not formally tested.

### Statistical analyses of MDA, GSA and NADP/NADPH

All assay values were log_2_-transformed prior to modelling. Statistical analyses were performed in R using the limma package. For each metabolite, a linear model was fit with diet (CD versus WD) and genotype as factors. Two sets of contrasts were tested. The first was to assess whether the WD effect in each genotype differed from that in the Null group, where contrasts were constructed as (WD – CD for each genotype) – (WD – CD for null). This evaluates whether the magnitude of the WD–CD change deviates significantly from the corresponding change in the Null genotype. The second was to test within-genotype WD versus CD, where WD samples were compared against the mean of CD samples of the same genotype to test whether the log_2_(WD/CD) ratio differed from zero (that is, whether the linear WD/CD fold change differed from 1). Moderated *t*-statistics and associated *P* values were obtained using empirical Bayes shrinkage (eBayes). Multiple testing correction was applied across assays using the Benjamini–Hochberg method, and adjusted *P* values (*q* values) are reported.

### Reporting summary

Further information on research design is available in the Nature Portfolio Reporting Summary linked to this article.

## Supplementary information

Supplementary Information Supplementary Fig. 1, including legend and source file for the figure, and Supplementary Table 1.

Reporting Summary

Supplementary Data 1 scPhenomics data.

Supplementary Data 2 Proteomics data.

## Source data

Source Data Extended Fig. 2 Unprocessed western blots and/or gels, statistical source data each figure panel appear as separate tab in the Excel file.

Source Data Extended Fig. 3 Unprocessed western blots and/or gels, statistical source data each figure panel appear as separate tab in the Excel file.

Source Data Extended Fig. 7 Unprocessed western Blots and/or gels, Statistical Source Data each figure panel appear as separate tab in the Excel file

Source Data Extended Fig. 8 Unprocessed western blots and/or gels, statistical source data each figure panel appear as separate tab in the Excel file.

Source Data Extended Fig. 9 Unprocessed western blots and/or gels, statistical source data each figure panel appear as separate tab in the Excel file.

Source Data Fig. 1 Unprocessed western blots and/or gels, statistical source data each figure panel appear as separate tab in the Excel file.

Source Data Fig. 4 Unprocessed western blots and/or gels, statistical source data each figure panel appear as separate tab in the Excel file.

Source Data Fig. 5 Unprocessed western blots and/or gels, statistical source data each figure panel appear as separate tab in the Excel file.

Source Data Fig. 6 Unprocessed western blots and/or gels, statistical source data each figure panel appear as separate tab in the Excel file.

Source Data Fig. 7 Unprocessed western blots and/or gels, statistical source data each figure panel appear as separate tab in the Excel file.

## Data Availability

The mass spectrometry dataset has been deposited in the MassIVE database under accession code MSV000093282. Microscopy datasets are available via figshare at 10.6084/m9.figshare.31017523 (ref. ^57^) and 10.6084/m9.figshare.31017544 (ref. ^58^). The remaining data are available in the Article, the Supplementary Information, Supplementary Data 1 and 2 and the Source Data file. For any additional information, please contact the corresponding author. Source data are provided with this paper.

## Code availability

scPhenomics code is available here: https://github.com/CCRMicroscopyCore/kangs.
