# Supplementary material for: PLIN5 phosphorylation orchestrates mitochondria lipid-droplet coupling to control hepatic lipid flux and steatosis
Source: Nat Metab. 2026 Mar 23;8(3):587–603. doi: 10.1038/s42255-026-01476-1 (PMC13031124; doi:10.1038/s42255-026-01476-1)
Supplement: Supplementary file 1 — Supplementary Fig. 1, including legend and source file for the figure, and Supplementary Table 1. [file 42255_2026_1476_MOESM1_ESM.pdf]

nature metabolism

Supplementary information

https://doi.org/10.1038/s42255-026-01476-1

PLIN5 phosphorylation orchestrates mitochondria lipid-droplet coupling to control hepatic lipid flux and steatosis

In the format provided by the authors and unedited

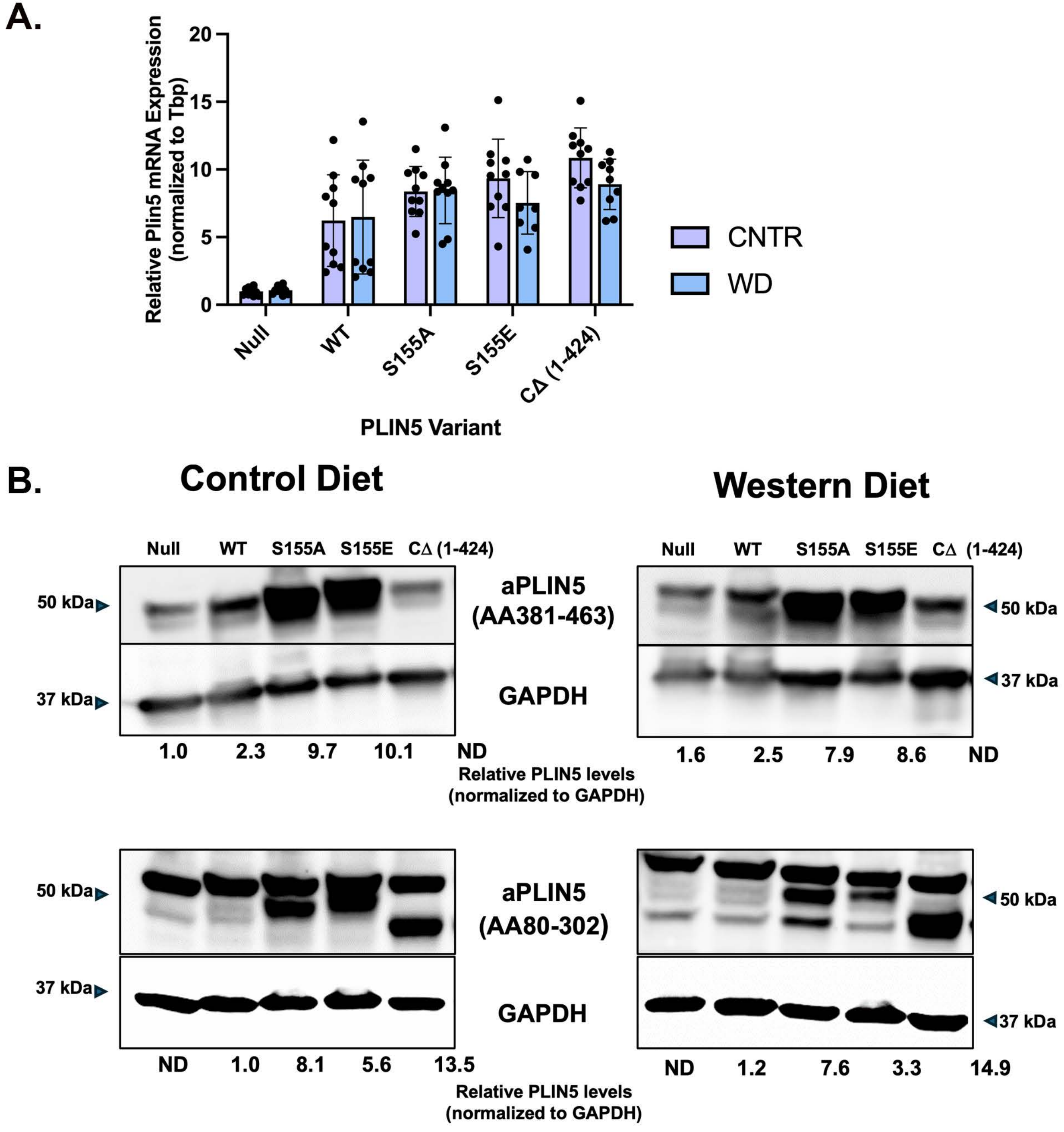

Supplementary Figure 1. In vivo overexpression of PLIN5

Supplement Figure 1. ***In vivo*** **overexpression of PLIN5. (A)** Relative Plin5 mRNA expression in mouse livers is presented as the mean ± SEM from n=10 mice. **(B)** Representative immunoblot and quantification of PLIN5 protein levels from one mouse per group. An antibody generated against the human PLIN5 carboxy terminus (upper panel) detects endogenous and exogenous full-length PLIN5 but does not detect the CΔ (1-424) variant. PLIN5 levels are relative to endogenous in the Null CNTR mouse. An antibody generated against amino acids within the amino terminus of human PLIN5 (lower panel) detects all forms of exogenous PLIN5 but does not detect endogenous levels in Null CNTR mice. PLIN5 levels are relative to WT exogenous levels in CNTR mice. Full-length PLIN5 migrates at 50 kDa, and the CΔ (1-424) PLIN5 migrates below 50 kDa. An intense non-specific >50kDa band is present in all samples, and a <50kDa band of undetermined origin is seen in WD-fed mouse samples.

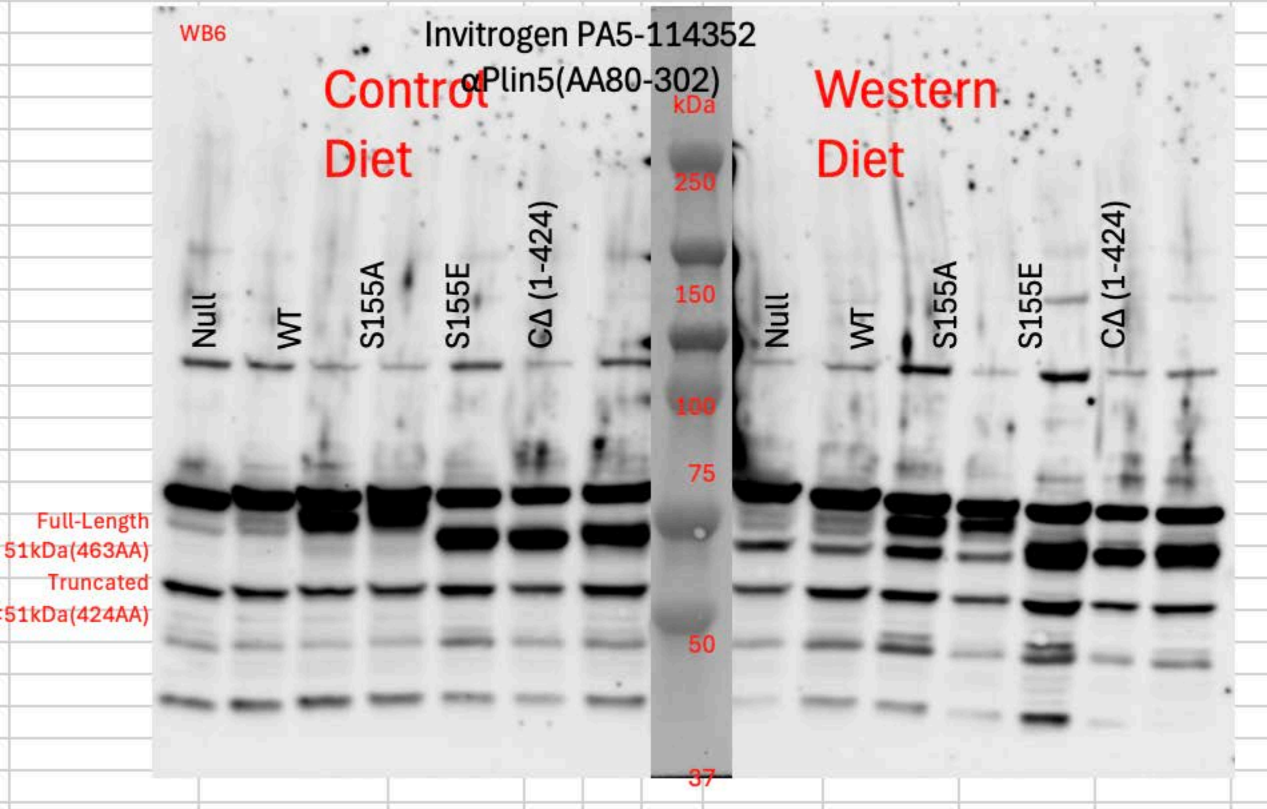
WB6
Invitrogen PA5-114352
αPlin5(AA80-302)
Control Diet
Western Diet
kDa
250
150
100
75
50
37
Null
WT
S155A
S155E
CΔ (1-424)
Null
WT
S155A
S155E
CΔ (1-424)
Full-Length
51kDa(463AA)
Truncated
51kDa(424AA)

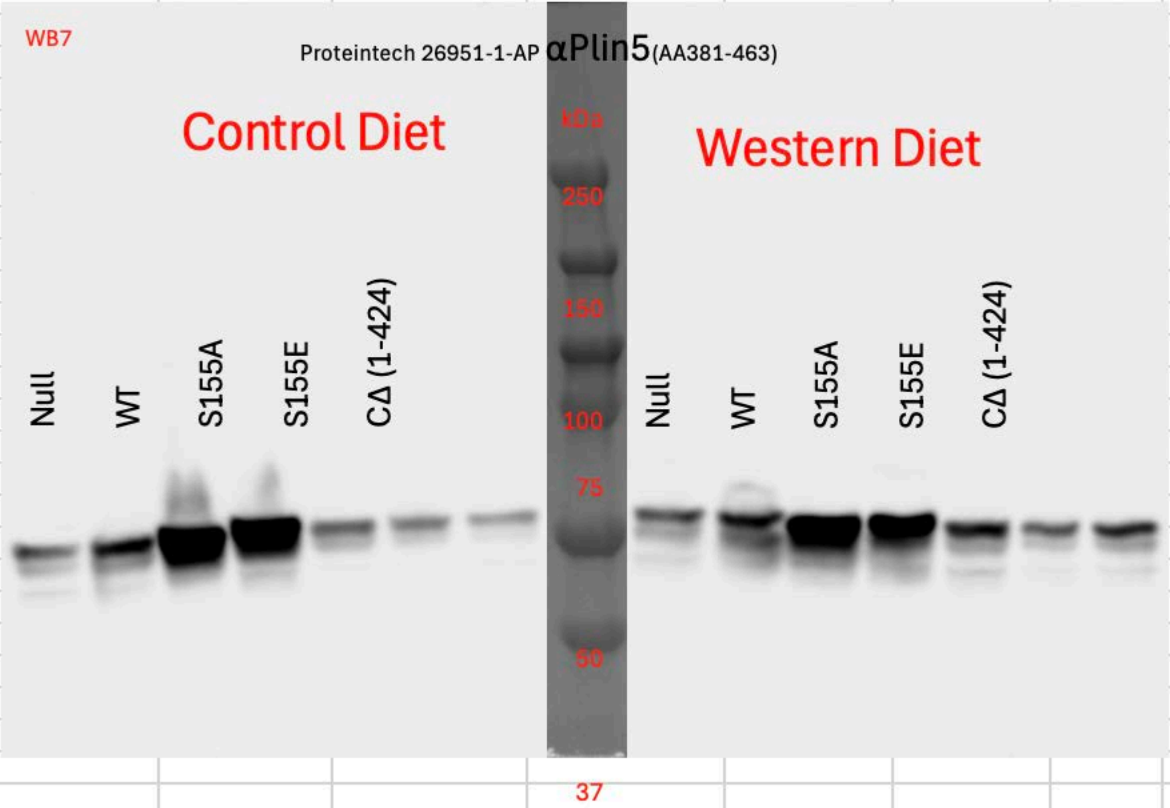
WB7
Proteintech 26951-1-AP αPlin5(AA381-463)
Control Diet
Western Diet
kDa
250
150
100
75
50
37
Null
WT
S155A
S155E
CΔ (1-424)
Null
WT
S155A
S155E
CΔ (1-424)

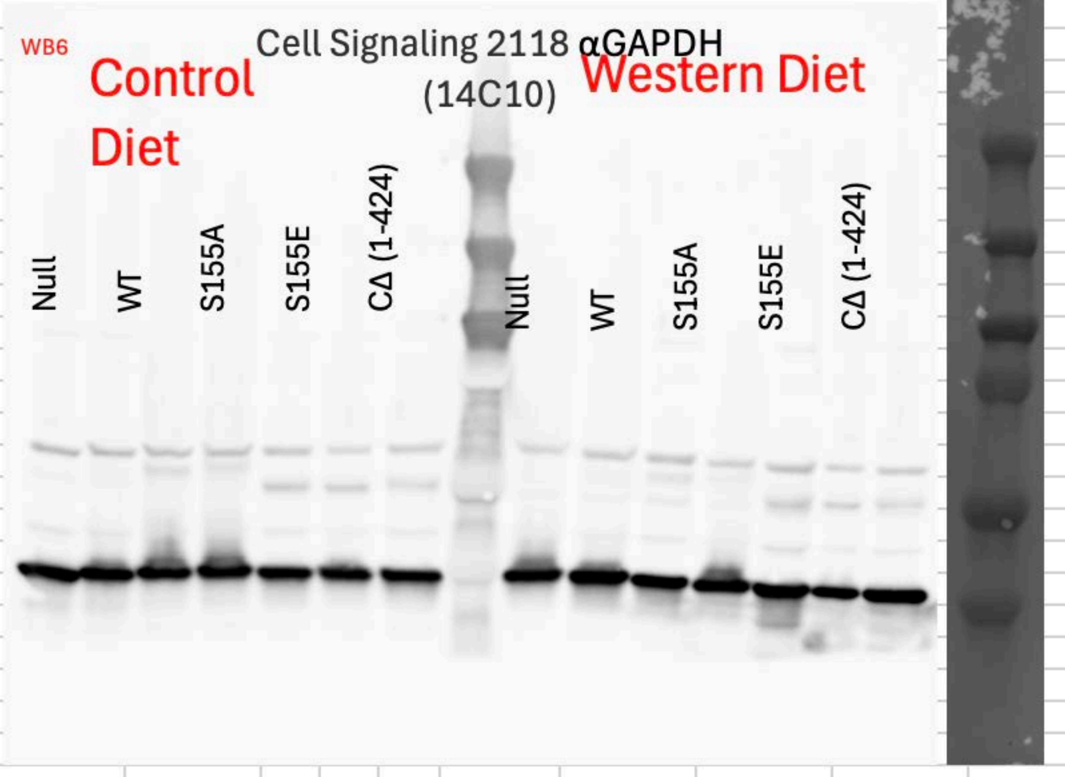
WB6
Cell Signaling 2118 αGAPDH
(14C10)
Control
Diet
Western Diet
Null
WT
S155A
S155E
CΔ (1-424)
Null
WT
S155A
S155E
CΔ (1-424)

NPS 2024-11-06 13h39m36s

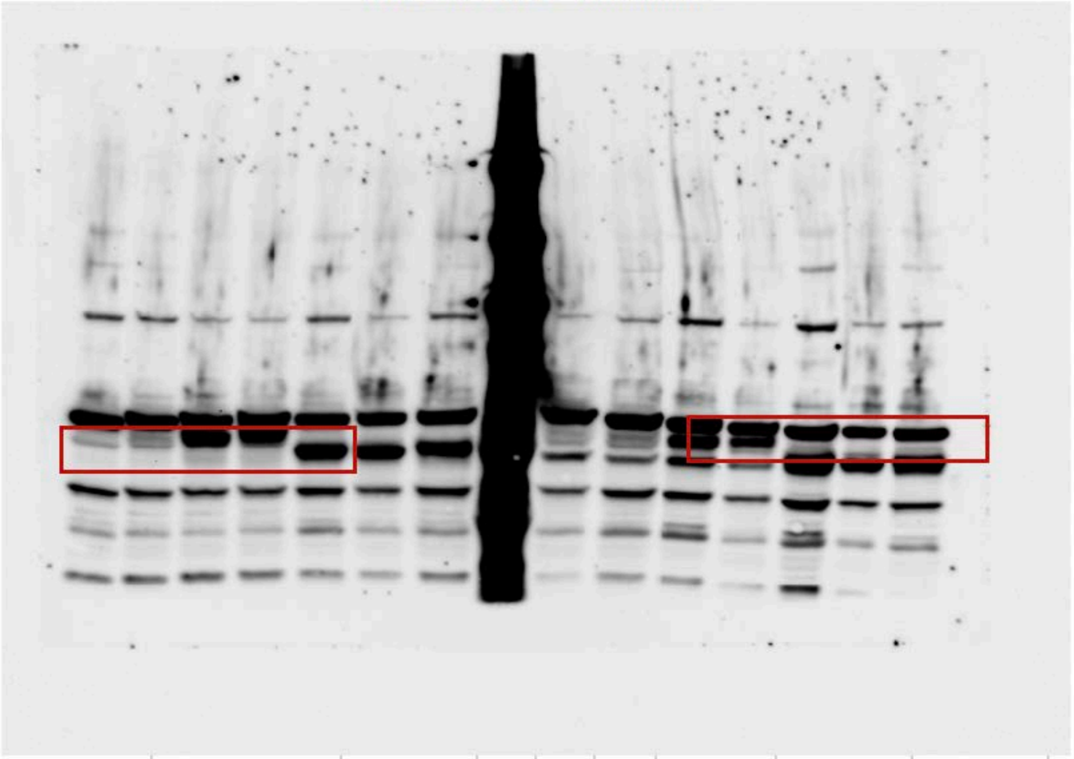

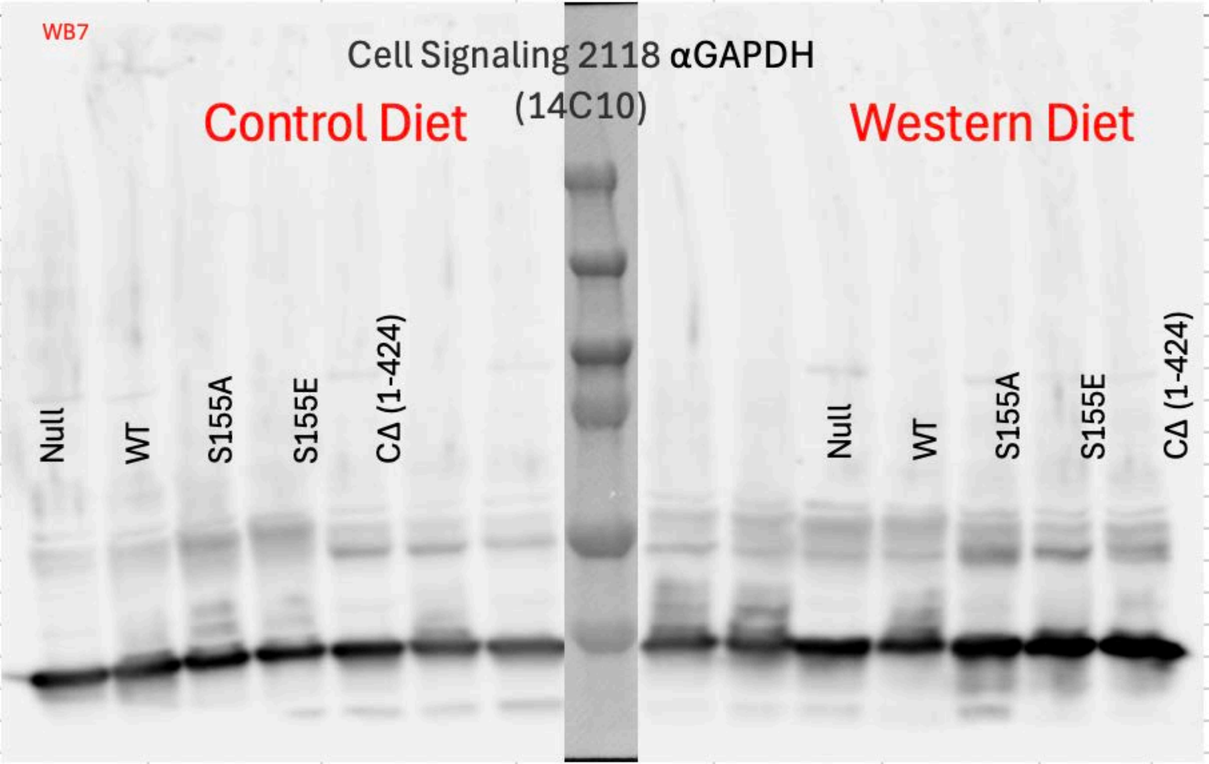
WB7
Cell Signaling 2118 αGAPDH
(14C10)
Control Diet
Western Diet
Null
WT
S155A
S155E
CΔ (1-424)
Null
WT
S155A
S155E
CΔ (1-424)

NPS 2024-11-06 13h39m36s			Invitrogen PA5-114352 αPlin5(AA80-302)								
No.	Volume (Int)	Adj. Vol. (Int)	Mean Bkgd. (Int)	Abs. Quant.	Rel. Quant.	# of Pixels	Min. Value (Int)	Max. Value (Int)	Mean Value (Int)	Std. Dev.	Area (mm2)
1	2,035,540.00	545,869.20	3,370.30	N/A	N/A	442	1,125.00	7,416.00	4,605.29	1,768.40	8.451931
2	3,099,218.00	883,084.12	5,013.88	N/A	N/A	442	1,373.00	10,855.00	7,011.81	2,520.32	8.451931
3	13,667,740.00	6,988,560.73	15,111.27	N/A	N/A	442	2,661.00	57,665.00	30,922.49	14,083.56	8.451931
4	12,070,028.00	4,999,976.41	15,995.59	N/A	N/A	442	2,647.00	49,724.00	27,307.76	13,898.97	8.451931
5	17,218,114.00	12,859,660.24	9,860.76	N/A	N/A	442	2,948.00	65,535.00	38,955.01	19,624.04	8.451931
8	2,951,964.00	799,685.59	4,869.41	N/A	N/A	442	1,556.00	9,854.00	6,678.65	1,877.54	8.451931
9	4,072,977.00	1,359,642.73	6,138.77	N/A	N/A	442	2,658.00	12,741.00	9,214.88	2,261.57	8.451931
10	12,741,054.00	8,437,489.43	9,736.57	N/A	N/A	442	1,856.00	65,535.00	28,825.91	15,455.68	8.451931
11	6,646,438.00	3,235,749.51	7,716.49	N/A	N/A	442	1,321.00	33,007.00	15,037.19	9,214.62	8.451931
12	19,391,330.00	13,136,588.00	14,151.00	N/A	N/A	442	2,002.00	65,535.00	43,871.79	17,835.17	8.451931

NPS 2024-11-07 11h15m55s			Cell Signaling 2118 αGAPDH (14C10)								
No.	Volume (Int)	Adj. Vol. (Int)	Mean Bkgd. (Int)	Abs. Quant.	Rel. Quant.	# of Pixels	Min. Value (Int)	Max. Value (Int)	Mean Value (Int)	Std. Dev.	Area (mm2)
1	7,922,854.00	4,812,792.76	5,253.48	N/A	N/A	592	1,027.00	25,489.00	13,383.20	6,711.57	11.32023
2	8,282,317.00	4,142,552.49	6,992.85	N/A	N/A	592	2,528.00	23,419.00	13,990.40	5,727.91	11.32023
3	7,977,966.00	4,036,612.98	6,657.69	N/A	N/A	592	1,823.00	22,729.00	13,476.29	5,043.72	11.32023
4	7,897,695.00	4,155,523.07	6,321.24	N/A	N/A	592	2,034.00	21,666.00	13,340.70	5,065.27	11.32023
5	7,000,574.00	4,457,912.47	4,295.04	N/A	N/A	592	1,908.00	21,635.00	11,825.29	5,801.53	11.32023
8	7,772,351.00	4,219,436.09	6,001.55	N/A	N/A	592	1,305.00	22,603.00	13,128.97	5,573.32	11.32023
9	9,165,508.00	5,103,882.11	6,860.85	N/A	N/A	592	2,157.00	24,402.00	15,482.28	5,759.73	11.32023
10	8,028,423.00	5,220,497.04	4,743.12	N/A	N/A	592	1,175.00	26,422.00	13,561.53	7,438.63	11.32023
11	8,557,636.00	4,649,574.91	6,601.45	N/A	N/A	592	1,074.00	24,811.00	14,455.47	5,802.50	11.32023
12	7,920,551.00	4,133,892.96	6,396.38	N/A	N/A	592	1,066.00	28,894.00	13,379.31	8,430.78	11.32023

WB6	Mouse#	PLIN5 variant	Adj. Vol. (Int)		Rel Abundance	
			Plin5	GAPDH	Plin5/GAPDH	
Control Diet	5	Null	545869.20	4812792.76	0.11	
	7	WT	883084.12	4142552.49	0.21	1.00
	28	S155A	6988560.73	4036612.98	1.73	8.12
	43	S155E	4999976.41	4155523.07	1.20	5.64
	25	AA 1-424	12859660.24	4457912.47	2.88	13.53
Western Diet	14	Null	799685.59	4219436.09	0.19	
	18	WT	1359642.73	5103882.11	0.27	1.25
	39	S155A	8437489.43	5220497.04	1.62	7.58
	52	S155E	3235749.51	4649574.91	0.70	3.26
	35	AA 1-424	13136588.00	4133892.96	3.18	14.91

NPS 2024-11-07 11h15m55s

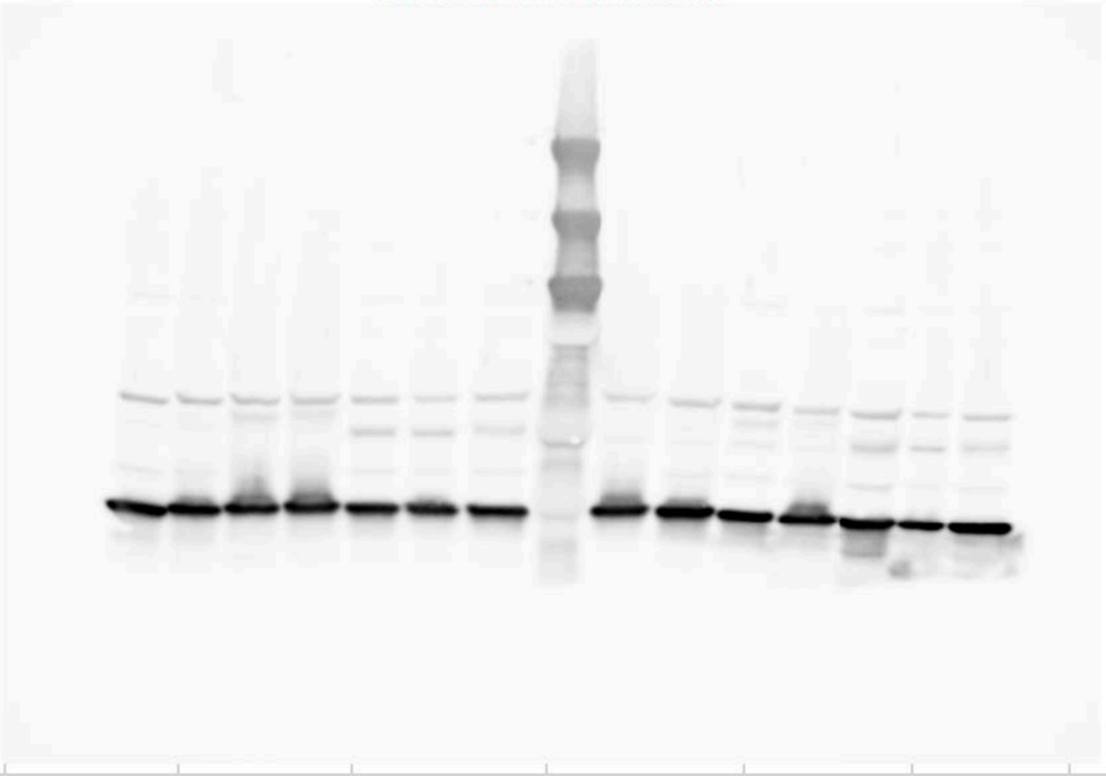

NPS 2024-11-13 13h51m05s

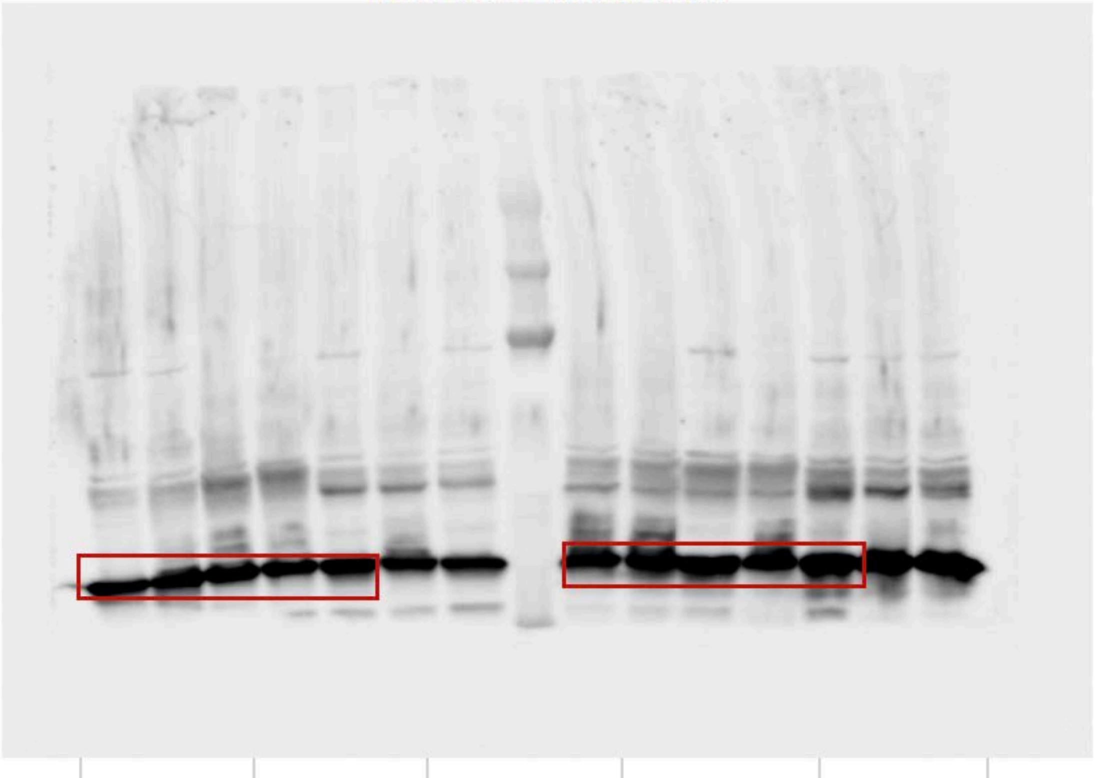

NPS 2024-11-06 14h07m18s

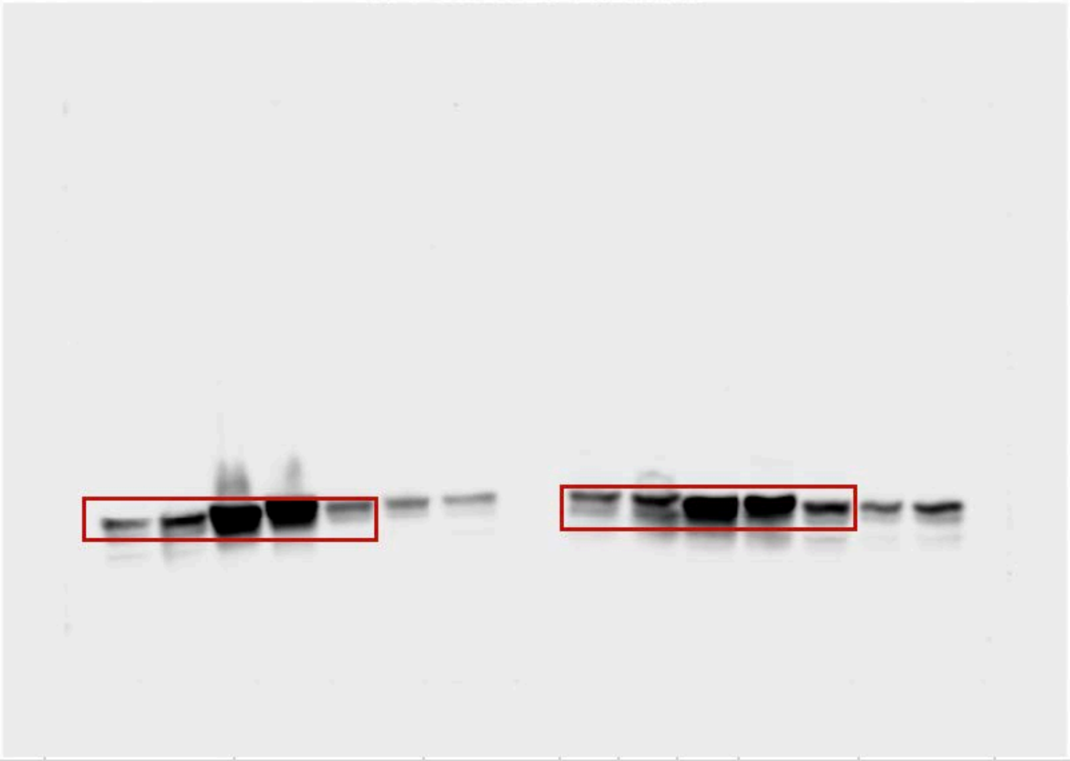

NPS 2024-11-06 14h07m18s			Proteintech 26951-1-AP αPlin5(AA381-463)								
No.	Volume (Int)	Adj. Vol. (Int)	Mean Bkgd.	Abs.	Rel.	# of	Min. Value	Max. Value	Mean Value	Std. Dev.	Area (mm2)
1	3,232,461.00	2,155,731.37	1,809.63	N/A	N/A	595	744	16,226.00	5,432.71	3,860.52	11.377599
2	6,670,683.00	3,897,211.70	4,661.30	N/A	N/A	595	815	24,544.00	11,211.23	6,448.77	11.377599
3	27,638,404.00	19,932,560.72	9,173.62	N/A	N/A	840	1,262.00	65,535.00	32,902.86	20,638.88	16.062492
4	26,008,073.00	20,629,759.56	6,402.75	N/A	N/A	840	1,363.00	65,535.00	30,961.99	19,456.20	16.062492
5	3,599,938.00	2,239,938.79	2,285.71	N/A	N/A	595	835	12,504.00	6,050.32	3,361.11	11.377599
8	4,487,177.00	2,871,575.70	2,715.30	N/A	N/A	595	1,155.00	16,808.00	7,541.47	3,390.05	11.377599
9	6,240,127.00	3,048,299.08	5,364.42	N/A	N/A	595	678	24,019.00	10,487.61	6,469.03	11.377599
10	24,576,459.00	18,008,753.75	7,818.70	N/A	N/A	840	3,603.00	65,535.00	29,257.69	17,102.31	16.062492
11	18,676,721.00	15,234,752.15	4,097.58	N/A	N/A	840	1,207.00	65,535.00	22,234.19	15,840.75	16.062492
12	5,463,930.00	3,821,079.91	2,761.09	N/A	N/A	595	659	22,519.00	9,183.08	6,715.12	11.377599

NPS 2024-11-13 13h51m05s			Cell Signaling 2118 αGAPDH (14C10)								
No.	Volume (Int)	Adj. Vol. (Int)	Mean Bkgd. (Int)	Abs. Quant.	Rel. Quant.	# of Pixels	Min. Value	Max. Value	Mean	Std. Dev.	Area
1	13,859,036.00	9,040,955.20	6,502.13	N/A	N/A	741	1,673.00	48,075.00	18,703.15	10,739.87	14.16941
2	13,202,737.00	7,075,391.36	9,725.95	N/A	N/A	630	1,041.00	44,237.00	20,956.73	9,890.70	12.04687
3	13,862,977.00	8,642,991.73	8,285.69	N/A	N/A	630	1,633.00	45,856.00	22,004.73	11,957.24	12.04687
4	12,571,830.00	8,576,788.09	6,341.34	N/A	N/A	630	1,868.00	44,556.00	19,955.29	11,732.65	12.04687
5	13,469,752.00	8,957,858.09	7,161.74	N/A	N/A	630	2,305.00	42,801.00	21,380.56	10,689.20	12.04687
8	13,331,558.00	7,674,980.13	7,633.71	N/A	N/A	741	1,211.00	34,072.00	17,991.31	7,308.17	14.16941
9	13,532,527.00	5,212,094.09	13,207.04	N/A	N/A	630	3,476.00	34,850.00	21,480.20	6,706.71	12.04687
10	17,414,175.00	9,568,880.73	10,587.44	N/A	N/A	741	2,280.00	45,494.00	23,500.91	12,980.86	14.16941
11	14,128,795.00	7,463,091.45	10,580.48	N/A	N/A	630	2,526.00	39,148.00	22,426.66	9,821.10	12.04687
12	19,938,094.00	11,227,503.15	11,755.18	N/A	N/A	741	2,913.00	51,718.00	26,907.01	12,442.58	14.16941

WB7	Mouse#	PLIN5 variant	Adj. Vol. (Int)		Rel Abundance	
			Plin5 Cterm	GAPDH	Plin5/GA	
Control Diet	5	Null	2155731.37	9040955.20	0.24	1.00
	7	WT	3897211.70	7075391.36	0.55	2.31
	28	S155A	19932560.72	8642991.73	2.31	9.67
	43	S155E	20629759.56	8576788.09	2.41	10.09
	25	AA 1-424	2239938.79	8957858.09	0.25	
Western Diet	14	Null	2871575.70	7674980.13	0.37	1.57
	18	WT	3048299.08	5212094.09	0.58	2.45
	39	S155A	18008753.75	9568880.73	1.88	7.89
	52	S155E	15234752.15	7463091.45	2.04	8.56
	35	AA 1-424	3821079.91	11227503.15	0.34	

#	Steatosis	Inflammation	Fibrosis
1	Minimal	None	None
2	None	None	None
3	None	None	None
4	Mild	Minimal	None
5	None	Mild	None
6	None	None	None
7	None	Minimal	None
8	Mild	Minimal	None
9	Minimal	Minimal	None
10	Mild	Mild	None
11	None	Mild	None
12	None	None	None

Supplementary Table 1: Histopathological summary of 12 healthy liver samples. Color coding indicates grouping based on the degree of steatosis.

Supplementary Table 2:

Antibodies	Source	Clone	Identifier	Dilution
APC anti-mouse CD73 antibody	Biolegend	TY/11.8	127210	1:150
Alexa Fluor® 594 anti-mouse/human CD324 (E-Cadherin) Antibody	Biolegend	DECMA-1	147306	1:100
Anti-mouse IgG, HRP-linked Antibody	Cell Signaling	NA	3662S	1:2500
Anti-rabbit IgG, HRP-linked antibody	Cell Signaling	NA	7076S	1:10000
Cyclophilin B (D1V5J) Rabbit monoclonal antibody	Cell Signaling	D1V5J	43603S	1:1000
Phospho-HSL (Ser565) Antibody	Cell Signaling	NA	4137S	1:1000
β-Actin (13E5) Rabbit monoclonal antibody	Cell Signaling	13E5	4970S	1:1000
β-actin (8H10D10) Mouse monoclonal antibody	Cell Signaling	8H10D10	3700S	1:1000
β-tubulin (9F3) Rabbit monoclonal antibody	Cell Signaling	9F3	2128S	1:1000
Goat anti-Rabbit IgG (H+L) Cross-Absorbed Secondary	Invitrogen™	NA	A11011	1:400

Antibody, Alexa Fluor 568				
Alexa Fluor™ 568 Phalloidin	Invitrogen™	NA	A12380	1:100
Alexa Fluor™ 647 Phalloidin	Invitrogen™	NA	A22287	1:100
Perilipin 5 Polyclonal antibody	Proteintech	NA	26051-1-AP	1:2000
OXPAT Polyclonal Antibody	Invitrogen™	NA	PA5-114352	1:500
GAPDH	Cell Signaling	14C10	2118S	1:1000

**Dyes**		
Alexa Fluor™ 568 Phalloidin	Thermo Fisher	A12380
HSC LipidTOX™ Deep Red Neutral Lipid Stain, for cellular imaging	Thermo Fisher	H34477
**Buffers**		
RIPA Lysis buffer	Thermo Fisher	J62524-AE
Thermo Scientific™ Halt™ Protease and Phosphatase Inhibitor Cocktail (100X)	Thermo Fisher	78442
Prec Plus Protein Dual Color Standards	Bio-Rad	1610374
10X Tris/Glycine/SDS	Bio-Rad	1610732
2X Laemmli Sample Buffer	Bio-Rad	1610737
Clarity Western ECL Substrate	Bio-Rad	1705061

Phosphate Buffered Saline, pH 7.2, 1X	Quality Biological	111-056-101
Hanks' Balanced Saline pH 7.2, 1X	Thermo Fisher	14174-095
Acetic acid	Sigma Aldrich	A6283
Collagenase from Clostridium histolyticum	Sigma Aldrich	C5138
Krebs-Henseleit Buffer Modified	Sigma Aldrich	K3753
Calcium chloride solution	Sigma Aldrich	21115
Sodium bicarbonate	Sigma Aldrich	S6014
Ethylenediaminetetraacetic acid disodium salt solution	Sigma Aldrich	03690
HEPES solution	Sigma Aldrich	H0887
Fetal Bovine Serum	Thermo Fisher	26140079
TMTpro™ 16plex Label Reagent set	Thermo Fisher	A44520
HiSelect ™ Phosphopeptide enrichment kit	Thermo Fisher	A32992
High Select TM Phosphopeptide Enrichment kit	Thermo Fisher	A32993
Trypsin Platinum	Promega	VA9000
Urea	Sigma Aldrich	51457
Methanol	Sigma Aldrich	34860
Trifluoroacetic acid	Sigma Aldrich	302031
Acetonitrile solution	Sigma Aldrich	900686
**qPCR**		

Plin5	Thermo Fisher	Mm00508854_m1
Tbp	Thermo Fisher	Mm01277041_m1
Taqman Fast Advanced Master Mix	Thermo Fisher	4444963
High-Capacity RNA to cDNA kit	Thermo Fisher	4388950
RNeasy mini kit	Qiagen	74104
**Assay**		
Pierce™ BCA Protein Assay kit	Thermo Fisher	23225
Triglyceride (TG) Colorimetric assay kit	Thermo Fisher	EEA028
NADP/NADPH assay kit	Sigma	Sigma- MAK479
Non-esterified Free Fatty Acids (NEFA/FFA) Colorimetric Assay Kit	Elabscience	E-BC-K013-M
GSH-Glo™ Glutathione Assay	Promega	V6911
Lipid peroxidation assay kit	Abcam	ab233471
**Other**		
Pierce™ Peptide Desalting Spin Columns	Thermo Fisher	89851
Mini-Protein® TGX™ Precast Gels	Bio-Rad	4568084
Trans-Blot Turbo NC Transfer Packs	Bio-Rad	1704158

scPhenomics image analysis

Overview

Image analysis workflow for liver lobule quantification for scPhenomics analysis. Code associated with Kang S, et al. Nature Metabolism 2025.

Image segmentation and quantification

Image segmentation and quantifcation utilizes Python (v3.10). Custom Cellpose models were used for segmentation. Segmentation using GPU-enabled HPC clusters is recommended, but is not essential. For image visualization and verification, napari multi-dimensional image viewer was used, but other software such as FIJI can be substituted.

lobule_analysis_v2.ipynb

- Load image
  - Define actin, mitochondria, and lipid channels
- Segment cells using a combination of the actin and mitochondria channel
  - Manual-editing of cell labels
- Segment mitochondria
- Segment lipid
- Create an overlap map of mitochondria and lipids
- Create Euclidean distance map from the central and portal veins
  - Manually define the central vein and create EDT map
  - Manually define the portal vein and create EDT map
- Create Euclidean distance map based on organelles
  - Create EDT map based on mitochondria
  - Create EDT map based on lipids
- Quantification of cells, mitochondria, lipids and organelle overlap regions
  - Measure channel intensity, area, centroid position and geometric parameters.

Data analysis

Data analysis scripts utilize R (v.4.2)

PLIN5_analysis.R

- Load quantification data
- Calculate a relative central - portal vein distance for cells and organelles by normalizing to the maximum CV-PV distance
- Based on relative distance, data was binned into 12 regions R1 - R12, with R1 closer to PV and R12 closer to CV
