# Supplementary material for: PLIN5 phosphorylation orchestrates mitochondria lipid-droplet coupling to control hepatic lipid flux and steatosis
Source: Nat Metab. 2026 Mar 23;8(3):587–603. doi: 10.1038/s42255-026-01476-1 (PMC13031124; doi:10.1038/s42255-026-01476-1)
Supplement: Supplementary file 2 — Reporting Summary [file 42255_2026_1476_MOESM2_ESM.pdf]

nature portfolio

Corresponding author(s): Natalie Porat-Shliom

Last updated by author(s): 01/06/2026

Reporting Summary

Nature Portfolio wishes to improve the reproducibility of the work that we publish. This form provides structure for consistency and transparency in reporting. For further information on Nature Portfolio policies, see our Editorial Policies and the Editorial Policy Checklist.

Statistics

For all statistical analyses, confirm that the following items are present in the figure legend, table legend, main text, or Methods section.

n/a	Confirmed	
☐	☒	The exact sample size (*n*) for each experimental group/condition, given as a discrete number and unit of measurement
☐	☒	A statement on whether measurements were taken from distinct samples or whether the same sample was measured repeatedly
☐	☒	The statistical test(s) used AND whether they are one- or two-sided<br>*Only common tests should be described solely by name; describe more complex techniques in the Methods section.*
☒	☐	A description of all covariates tested
☐	☒	A description of any assumptions or corrections, such as tests of normality and adjustment for multiple comparisons
☐	☒	A full description of the statistical parameters including central tendency (e.g. means) or other basic estimates (e.g. regression coefficient) AND variation (e.g. standard deviation) or associated estimates of uncertainty (e.g. confidence intervals)
☐	☒	For null hypothesis testing, the test statistic (e.g. F, t, r) with confidence intervals, effect sizes, degrees of freedom and P value noted<br>*Give P values as exact values whenever suitable.*
☒	☐	For Bayesian analysis, information on the choice of priors and Markov chain Monte Carlo settings
☒	☐	For hierarchical and complex designs, identification of the appropriate level for tests and full reporting of outcomes
☐	☒	Estimates of effect sizes (e.g. Cohen's *d*, Pearson's *r*), indicating how they were calculated

Our web collection on statistics for biologists contains articles on many of the points above.

Software and code

Policy information about availability of computer code

Data collection	In each experiment, the instrument's software was used for data collection as described in Materials and methods and figure legends
Data analysis	Data analysis information is in the Materials and methods and figure legends. Common softwares include Microscopy analysis: FiJi and Imaris; FACS analysis: FlowJo; Statistics: Excel and Prism. The code developed is available:https://github.com/CCRMicroscopyCore/kangs.

For manuscripts utilizing custom algorithms or software that are central to the research but not yet described in published literature, software must be made available to editors and reviewers. We strongly encourage code deposition in a community repository (e.g. GitHub). See the Nature Portfolio guidelines for submitting code & software for further information.

Data

Policy information about availability of data

All manuscripts must include a data availability statement. This statement should provide the following information, where applicable:

- Accession codes, unique identifiers, or web links for publicly available datasets
- A description of any restrictions on data availability
- For clinical datasets or third party data, please ensure that the statement adheres to our policy

Data availability
The mass spectrometry dataset has been deposited in the MassIVE database under accession code MSV000093282 [https://massive.ucsd.edu/ProteoSAFe/static/massive.jsp]. Microscope images analysed are available: https://figshare.com/s/d148f89123a79770b37a

The remaining data are available within the Article, in the Supplementary Information, Supplementary Data 1-2, and in the Source Data file. For any additional information, please contact the corresponding author.
The remaining data are available within the Article, Supplementary table

Research involving human participants, their data, or biological material

Policy information about studies with human participants or human data. See also policy information about sex, gender (identity/presentation), and sexual orientation and race, ethnicity and racism.

Reporting on sex and gender	Sex and/or gender was not considered in the study design. Sex and/or gender of participants was determined based on self-reporting
Reporting on race, ethnicity, or other socially relevant groupings	*Please specify the socially constructed or socially relevant categorization variable(s) used in your manuscript and explain why they were used. Please note that such variables should not be used as proxies for other socially constructed/relevant variables (for example, race or ethnicity should not be used as a proxy for socioeconomic status).* *Provide clear definitions of the relevant terms used, how they were provided (by the participants/respondents, the researchers, or third parties), and the method(s) used to classify people into the different categories (e.g. self-report, census or administrative data, social media data, etc.)* *Please provide details about how you controlled for confounding variables in your analyses.*
Population characteristics	Human tissue was acquired with written informed consent under an NIH IRB-approved protocol (NCT01915225) during risk-reducing surgery performed on patients with germline mutation in the tumor suppressor CDH1. Mutations in CDH1 are a frequent cause of the hereditary diffuse gastric cancer (HDGC) syndrome, which is associated with gastric adenocarcinoma, lobular breast cancer, and cleft lip/palate. These patients undergo risk-reducing total gastrectomy, and some consent to a liver biopsy during the surgery. While these patients often have microscopic stage 1A cancers, none had deeply invasive or metastatic carcinoma in their livers.
Recruitment	*Describe how participants were recruited. Outline any potential self-selection bias or other biases that may be present and how these are likely to impact results.*
Ethics oversight	NIH IRB-approved protocol (NCT01915225)

Note that full information on the approval of the study protocol must also be provided in the manuscript.

Field-specific reporting

Please select the one below that is the best fit for your research. If you are not sure, read the appropriate sections before making your selection.

☒ Life sciences ☐ Behavioural & social sciences ☐ Ecological, evolutionary & environmental sciences

For a reference copy of the document with all sections, see nature.com/documents/nr-reporting-summary-flat.pdf

Life sciences study design

All studies must disclose on these points even when the disclosure is negative.

Sample size	All experiments were replicated at least three independent experiments. Covariates in mouse experiments are controlled through rigorous experimental design (randomization, blocking, standardization) and statistical analysis (including them as variables in models) to ensure that the observed outcomes are due to the independent variable (treatment) and not other influences. No statistical methods were used to pre-determine sample sizes, but our sample sizes are similar to those reported in previous publications 7, 53, 56, 57. Data distribution was assumed to be normal, but this was not formally tested.
Data exclusions	Outliers were excluded using Grubbs' Test in Prism
Replication	All experiments were replicated with similar trends.
Randomization	No randomization was used
Blinding	Investigators were blinded to group allocation during data collection and analysis

Reporting for specific materials, systems and methods

We require information from authors about some types of materials, experimental systems and methods used in many studies. Here, indicate whether each material, system or method listed is relevant to your study. If you are not sure if a list item applies to your research, read the appropriate section before selecting a response.

Materials & experimental systems

n/a	Involved in the study	
☐	☒	Antibodies
☒	☐	Eukaryotic cell lines
☒	☐	Palaeontology and archaeology
☐	☒	Animals and other organisms
☒	☐	Clinical data
☒	☐	Dual use research of concern
☒	☐	Plants

Methods

n/a	Involved in the study	
☒	☐	ChIP-seq
☐	☒	Flow cytometry
☒	☐	MRI-based neuroimaging

Antibodies

Antibodies used

APC anti-mouse CD73 antibody Biolegend 127210 1:150 clone TY/11.8
Alexa Fluor® 594 anti-mouse/human CD324 (E-Cadherin) clone DECMA-1
Antibody Biolegend 147306 1:100
Anti-mouse IgG, HRP-linked Antibody Cell Signaling 3662S 1:10000
Anti-rabbit IgG, HRP-linked antibody Cell Signaling 7076S 1:10000
Cyclophilin B (D1V5J) Rabbit monoclonal antibody Cell Signaling 43603S 1:1000 clone D1V5J
β-actin (8H10D10) Mouse monoclonal antibody Cell Signaling 3700S 1:1000 clone 13E5
β-tubulin (9F3) Rabbit monoclonal antibody Cell Signaling 2128S 1:1000 clone 9F3
Goat anti-Rabbit IgG (H+L) Cross-Absorbed Secondary Antibody, Alexa Fluor 568 InvitrogenTM A11011 1:400
Alexa FluorTM 568 Phalloidin InvitrogenTM A12380 1:100
Alexa FluorTM 647 Phalloidin InvitrogenTM A22287 1:100
Perilipin 5 Polyclonal antibody Proteintech 26051-1-AP 1:2000
OXPAT Polyclonal Antibody InvitrogenTM PA5-114352 1:500
GAPDH Cell Signaling 2118S 1:1000 clone 14C10

Validation

Antibodies were validated as indicated on the manufacturer's website

Animals and other research organisms

Policy information about studies involving animals; ARRIVE guidelines recommended for reporting animal research, and Sex and Gender in Research

Laboratory animals

Experiments were approved by the Institutional Animal Care and Use Committee of the National Cancer Institute
Experiments were approved by the Institutional Animal Care and Use Committee of the National Cancer Institute and in compliance with the Guide for the Care and Use of Laboratory Animals (National Institutes of Health publication 86-23, revised 1985). All experiments were conducted on ad libitum-fed on normal chow diet (NIH-31 Open Formula) or western diet (TD.120528, Envigo), four to ten weeks old C57BL/6J (strain# 000664) or mtDendra2 excised 20 (photo-activatable mitochondria; strain# 018397) male mice obtained from Jackson Laboratories.

Wild animals

Not used

Reporting on sex

Male mice were used in this study

Field-collected samples

Not available

Ethics oversight

Experiments were approved by the Institutional Animal Care and Use Committee of the National Cancer Institute comply with the Guide for the Care and Use of Laboratory Animals

Note that full information on the approval of the study protocol must also be provided in the manuscript.

Plants

Seed stocks

Report on the source of all seed stocks or other plant material used. If applicable, state the seed stock centre and catalogue number. If plant specimens were collected from the field, describe the collection location, date and sampling procedures.

Novel plant genotypes

Describe the methods by which all novel plant genotypes were produced. This includes those generated by transgenic approaches, gene editing, chemical/radiation-based mutagenesis and hybridization. For transgenic lines, describe the transformation method, the number of independent lines analyzed and the generation upon which experiments were performed. For gene-edited lines, describe the editor used, the endogenous sequence targeted for editing, the targeting guide RNA sequence (if applicable) and how the editor was applied.

Authentication

Describe any authentication procedures for each seed stock used or novel genotype generated. Describe any experiments used to assess the effect of a mutation and, where applicable, how potential secondary effects (e.g. second site T-DNA insertions, mosiacism, off-target gene editing) were examined.

Flow Cytometry

Plots

Confirm that:

☐ The axis labels state the marker and fluorochrome used (e.g. CD4-FITC).

☐ The axis scales are clearly visible. Include numbers along axes only for bottom left plot of group (a 'group' is an analysis of identical markers).

☐ All plots are contour plots with outliers or pseudocolor plots.

☐ A numerical value for number of cells or percentage (with statistics) is provided.

Methodology

Sample preparation	Described in Materials and methods
Instrument	*Identify the instrument used for data collection, specifying make and model number.*
Software	*Describe the software used to collect and analyze the flow cytometry data. For custom code that has been deposited into a community repository, provide accession details.*
Cell population abundance	*Describe the abundance of the relevant cell populations within post-sort fractions, providing details on the purity of the samples and how it was determined.*
Gating strategy	*Describe the gating strategy used for all relevant experiments, specifying the preliminary FSC/SSC gates of the starting cell population, indicating where boundaries between "positive" and "negative" staining cell populations are defined.*

☐ Tick this box to confirm that a figure exemplifying the gating strategy is provided in the Supplementary Information.
